# Abstracts from the 7th International Jerusalem Conference on Health Policy: Jerusalem, Israel. 15-16 September 2019

**DOI:** 10.1186/s13584-019-0336-2

**Published:** 2019-09-10

## Track A: Data-driven care - realizing the promise

### Parallel 1

#### Abstract 168 Population health care for children with ongoing conditions

##### Ingrid Wolfe^1^, Claire Lemer^2^, Julia Forman^3^, James Newham^3^, Raghu Lingam^5^

###### ^1^King’s College London; Evelina London Children’s Healthcare, UK; ^2^Evelina London Children’s Healthcare, UK; ^3^King’s College London, UK; ^4^University of New South Wales, Australia

####### **Correspondence:** Ingrid Wolfe

**Background:** Children and young people (CYP) with ongoing physical health conditions such as asthma are affected by mental health and social conditions. The current healthcare model designed for acute episodic illness delivers variable and poor outcomes, and demand is rising unsustainably.

We present a population approach to healthcare delivering high quality preventive and responsive biopsychosocial care as part of clinical-academic health systems strengthening initiative, using data to inform a population registry for improving direct patient care and system intelligence for improving services.

**Study Question:** Can data-driven population health management improve health outcomes and equity among children with ongoing conditions?

**Methods:** An opportunistic cluster randomised control trial, measuring health, healthcare quality, and healthcare use between intervention and control, and before-after implementation. Population approach to case finding and improving equity of access to care will be assessed by comparing coverage and uptake rates with prevalence of health and population socio-economic conditions. Estimates of ED attendances prevented by comparing attendances in the quarter before and after receiving CYPHP care, using routine hospital administrative data.

**Results:** The first wave of active case finding reached 90% eligible population; 11% participated in early intervention. Demographic and socio-economic characteristics of participants suggest this approach enhances equity of access to care; a high proportion of CYP from ethnic minority families and living in deprived conditions. Early results suggest improved healthcare quality and reductions in ED use among patients with asthma, epilepsy, or constipation. We estimate 288 fewer ED contacts per asthma 100 patients, per year, 120 for epilepsy, and 60 for constipation.

**Conclusions:** A population approach to biopsychosocial care can improve early intervention and care among CYP with ongoing conditions.

**Health Policy Implications:** Population health approaches to care can improve health, healthcare quality, and health service sustainability. Clinical academic partnerships and learning health systems are a successful means of strengthening health systems and improving health.

#### Abstract 75 Repurposing medications by using maccabi health service electronic database

##### Gideon Koren

###### MK&M Research & Innovation Institute, IL

**Background:** Repurposing of existing drugs for new indications can save years of research and tremendous resources.

**Study Question:** Can we test a new repurposing model and create an algorithm by utilizing machine learning of big data to identify the potential role of concomitant drugs taken by hypertensive and diabetic Type 2 patients.

**Methods:** We applied machine learning techniques to identify concomitant drugs not taken for hypertension or diabetes which may contribute to lowering blood pressure or improving glucose control.

Success in controlling blood pressure was achieving blood pressure below 140/90 within 90 days of therapy, and success in diabetes control was defined as achieving HgA1c levels <6.5 between 90 to 365 days following diagnosis and initiating treatment.

**Results:** Among numerous concomitant drugs taken by hypertensive patients, statins and protein pump inhibitors (PPI) significantly improved blood pressure. For Type 2 diabetic patients, alpha 1 blocking drugs were the only group of medications to significantly improve the success rate of glucose control. These effects of statins, PPI and alpha 1 blockers shown by us have been recently documented in animal models and small human studies, validating this new innovative method.

**Conclusions:** Machine learning of big data is a novel method to identify effective repurposing of medications already on the market for new indications. Our model has been validated by contrasting these discoveries with recent animal and human studies.

**Health Policy Implications:** In the case of alpha 1 blockers, because this class of medications is widely used in men with benign prostate hyperplasia at age groups with increased rates of type 2 diabetes, this finding is of potential clinical and health policy significance.

#### Abstract 277 India’s national family health survey (2015-16) big data awaits advanced analytics and artificial intelligence applications towards strengthening public health outreach and outcomes

##### Kandarp Talati^1^, Geetika Madan-Patel^2^, Priya Swaminarayan^2^

###### ^1^Foundation for Diffusion of Innovations, India; ^2^Parul University, Limda, Vadodara, India

####### **Correspondence:** Kandarp Talati

**Background:** India undertook National Family Health Survey (NFHS-4;2015-16) almost after a decade. For the first time data collection was done at district-level with sample size of 601,509 households. Data is freely available on Demographic and Health Survey (DHS) website and Government of India (GoI) has also published factsheets (district/state/ national-level), national and state reports highlighting key findings from NFHS-4 data. Currently NFHS-5(2018-19) is underway and still, there is dearth of credible research output from NFHS-4.

**Study Question:** What opportunities does Artificial Intelligence (AI) provide with NFHS-4 and similar open-access big data for transforming public health landscape in resource-constrained settings?

**Methods:** Authors with qualifications in public health, community medicine and computer science reviewed recent literature and discussed potential opportunities for AI in NFHS-4 based on their individual experience and domain expertise.

**Results:** NFHS-4 has sensational media coverage with 6,000+ articles. On contrary, PubMed has <20 research papers exploring NFHS-4 dataset. Availability of online open-access AI-powered analytics portal could empower key players to identify critical public health challenges, ranging from malnutrition to malignancies, in their population catchment from NFHS-4/DHS data. The portal could help identify key social and other determinants of health and predict future trends based on available data and suggest targeted interventions which may be specific to region/locality under consideration. The portal could be programmed as an expert system to suggest context-specific interventions and guide uniform data collection for tracking intervention fidelity, its evaluation and real-time decision support.

**Conclusions:** AI-powered analytics support to mine NFHS/DHS data could empower academic scholars and local institutions to pursue need-based research and/or public health service delivery.

**Health Policy Implications:** Advances in data analysis can be leveraged with AI applications for evidence-based decision making and problem-solving by government, non-government and private health service providers. Public leadership in such portal may facilitate data collection, aggregation and validation from different sources and guide future strategies for NFHS/DHS based on emerging trends.

#### Abstract 24 Burden of mortality due to ambient fine particulate air pollution (PM2.5) in Israel

##### Alina Vodonos^1^, Itai Kloog^2^, Joel Schwartz^1^

###### ^1^Harvard T.H. Chan, US; ^2^Ben-Gurion University of the Negev, IL

####### **Correspondence:** Alina Vodonos

**Background:** Exposure to fine particulate matter (<2.5 mm in aerodynamic diameter, PM2.5) is a major source of reduced lifespan worldwide. Despite recent reduction of air pollution levels in Israel, adverse health effects of PM2.5 remain a regulatory and public health concern as recent studies have shown effects at lower concentrations. Many health impact assessments produce national or international estimates of the health burden.

**Study Question:** In this study, we present the estimates of the effect of PM2.5 on mortality highlighting differential effects by local districts in Israel.

**Methods:** Health impact assessments for 2005, 2010 and 2015 were performed using a validated hybrid satellite-based spatiotemporal model for PM2.5 combined with population data and district level mortality rates. Relative risks were derived from a previously published meta-analysis of association between long-term exposure to PM2.5 and mortality. Four scenarios were considered; a decrease of the air pollutant levels by 10% and 20%, a decrease to the World Health Organization (WHO) air quality guidelines, and a decrease to the background level found in the absence of emissions from transportation.

**Results:** The mean of PM2.5 in 2005, 2010 and 2015 was 18.8±7.2, 21.99±16.7 and 20.48±23.8 μg/m3, respectively. All location exceeded the WHO air quality guidelines of 10 μg/m3. In 2015, the absence of emissions from transportation would postpone 1,868 deaths. Complying with the WHO guidelines would postpone up to 4,452 deaths. The burden was unequally distributed, with the majority of premature deaths occurring in Haifa, Tel Aviv, and Hamerkaz districts.

**Conclusions:** High background levels together with anthropogenic pollution result in Israeli residents being exposed to a high concentration of PM2.5. Our study provides evidence of major health benefits expected from reducing the anthropogenic source of pollution by shifting to a low emission vehicles alternative.

**Health Policy Implications:** The estimates are expected to be useful to policymakers and others to craft more effective policies to mitigate the adverse impact of air pollution on the public’s health.

#### Abstract 259 Barriers to treatment in Israeli Arab minority adolescents with mental health problems: results from the Galilee study

##### Raida Daeem^1^, Ivonne Mansbach-Kleinfeld^2^, Ilana Farbstein^1^, Alan Apter^2^

###### ^1^Ziv Medical Center, IL; ^2^Schneider Medical Center for Children in Israel, IL

####### **Correspondence:** Raida Daeem

**Background:** The Galilee Study is the first study to assess mental health service needs among Israeli Muslim and Druze adolescents and their mothers.

**Study Question:** What are the structural and cultural barriers to help-seeking among the mothers considering seeking help for their children.

**Methods:** All 9^th^-grade students living in 5 towns representative of Muslim and Druze localities in northern Israel, were eligible for the study and 1639 (69.3%) obtained parental agreement and participated. The emotional or behavioral problem was assessed in the classroom using the Strengths and Difficulties Questionnaire. A total of 704 adolescent-mother dyads participated in the follow-up, and were interviewed at home, using the Development and Well Being Assessment inventory, the Composite Barriers to Help-Seeking Questionnaire, the General Health Questionnaire -12 and socio-demographic questions.

**Results:** More mothers of adolescents with a mental disorder than those without a mental disorder consulted a professional or school source (39.7% vs. 20.5%; χ2=45.636; p=<0.001). The most important barriers to help-seeking were those related to “Accessibility”, followed by barriers related to the belief that “Treatment is detrimental” and to the possibility of “Reprisal by authorities”. Barriers related to “Stigma” and “Distrust of professionals” had the lowest mean scores.

**Conclusions:** Structural barriers related to lack of access, were considered the main obstacle to help-seeking in this Israeli Arab minority population. Cultural barriers such as stigma were considered of secondary importance. The findings of the Galilee Study undermine the preconceptions and prejudice about service use among Arab minority in Israel and provide guidance on planning services for the minority Muslim and Druze populations in the coming decade.

**Health Policy Implications:** Structural barriers could be reduced by increasing the number of accessible public mental health clinics in the minority localities, a responsibility of the Ministry of Health and the HMOs. Information campaigns and psychoeducation for parents would help reduce other barriers to mental health treatment.

#### Abstract 130 The appropriate use of antipsychotics: supporting quality improvement in supportive living

##### Deborah Katz, Mollie Cole

###### Alberta Health Services, Canada

####### **Correspondence:** Deborah Katz

**Background:** This project aimed to address the appropriate use of antipsychotics in Supportive Living. Side effects of antipsychotics include agitation, confusion, falls, insomnia, sedation, increased risks of infection, strokes and cardiac events. Families, physicians and staff work together to investigate and trial alternate approaches to reduce agitation.

The Alberta Health Services Seniors Health Strategic Clinical Network initiative to reduce the use of Antipsychotics in Long Term Care Facilities continues to achieve success. The existence and publication by the Canadian Institute for Health Information (CIHI) of the interRAI Resident Assessment Instrument (RAI-MDS 2.0) Quality Indicator (QI) - Percent of Residents on Antipsychotics without a Diagnosis of Psychosis provides a metric that can accessed and reported quarterly. By acquiring and integrating data from Emergency, Inpatients, Physician Claims, Pharmacy Information Network and the Resident Assessment Instrument-Home Care (RAI-HC), a similar metric can be developed to monitor and report on the use of Antipsychotics in Supportive Living environments.

**Study Question:** Can existing data be leveraged to create a metric to support improvements in inter-professional practice and care for residents and families by spreading the Appropriate use of Antipsychotics initiatives from Long Term Care Facilities to Supportive Living environments in Alberta?

**Methods:** Data sources include Home Care, RAI-HC, National Ambulatory Care Reporting System, Inpatient Discharge Abstract Database, Physician Claims and the Pharmacy Information Network. Data analytic, visualization and liberation tools include Oracle, SQL Developer, Excel and Tableau.

**Results:** Referencing the RAI-MDS 2.0 QI, similar core elements have been acquired and a comparable antipsychotic measure for community living clients has been generated.

**Conclusions:** This work allows for measuring, monitoring and reporting on the use of Antipsychotics for individuals residing in Supportive Living environments in Alberta.

**Health Policy Implications:** These antipsychotic utilization measures are designed to support improvements in inter-professional practice and the quality of care delivered to residents and their families.

#### Abstract 146 Building big data from experience: a new model for prems collection and utilisation

##### Ilaria Corazza, Kendall Jamieson Gilmore, Manila Bonciani, Sabina De Rosis

###### Sant'Anna School of Advanced Studies, Pisa, Italy

####### **Correspondence:** Ilaria Corazza

**Background:** Patient-reported experience measures (PREMs) can help the design and management of healthcare services, and inform policymaking. However, the experience is typically measured using standard closed-ended questions, collected only periodically and unsystematically. This dearth of data is particularly problematic in pediatric settings due to exacerbated information and power asymmetries.

**Study Question:** How can healthcare providers make use of new technologies and analytical techniques to enable the systematic and continuous collection and utilisation of pediatric PREMs?

**Methods:** This study describes the cases of Meyer Hospital (Florence) and Children’s Clinical University Hospital (Riga) that, from December 2018, adopted a digital PREMs survey. The questionnaire was developed by hospital managers and physicians, collaborating with researchers from the MeS Laboratory - Sant’Anna School of Advanced Studies (Pisa). It consists of open-ended and closed-ended questions, some of which are adopted from the pediatric Hospital Consumer Assessment of Healthcare Providers and Systems (HCAHPS). It can be answered directly by adolescent patients or by caregivers and includes a section specifically addressed to children. The questionnaire is administered digitally upon discharge to all enrolled patients. A web platform collects, analyses and illustrates data in aggregate and anonymous form to hospital staff in real time.

**Results:** This study sets out the development of a new pediatric PREMs questionnaire, plus a digital and automatic survey administration and data reporting system.

**Conclusions:** This model has several features which may be of interest to clinicians and administrators and can be replicated elsewhere: notably, inclusion of narrative sections, enabling greater richness of information; differential access for different staff groups and researchers through an online platform, enabling prompt use of data and possibilities for action; dual implementation in two sites in different settings, enabling comparison and shared learning.

**Health Policy Implications:** This approach to PREMs can provide professionals at all levels in healthcare systems with a novel source of insight to support quality improvements.

#### Abstract 61 Barriers to completing colonoscopy after a positive fecal occult blood test

##### Liora Valinsky^1^, Revital Azulay^1^, Einat Elran^2^, Fabienne Hershkowitz^1^, Natan Lederman^1^, Revital Kariv^2^, Anthony Heymann^1^

###### ^1^Meuhedet Healthcare Services, IL; ^2^Maccabi Healthcare Services, IL

####### **Correspondence:** Liora Valinsky

**Background:** Colorectal cancer leads to significant morbidity and mortality. Early detection and treatment are essential. Screening using fecal occult blood tests (FOBT) has increased significantly, but adherence to colonoscopy follow-up is suboptimal worldwide. Recently published papers have emphasized the increased risk of abstaining from colonoscopy after a positive FOBT.

**Study Question:** What are the barriers to colonoscopy following a positive FOBT at the level of the patient, physician, organization, and policymakers.

**Methods:** This mixed methods study was conducted at two health care organizations in Israel. The study included retrospective analyses of 45,281 50-74-year-old members with positive FIT's from 2010-2014, and a survey of 772 patients with positive FIT during 2015, with and without follow-up. The qualitative part of the study included focus groups with primary physicians and gastroenterologists and in-depth interviews with opinion leaders in healthcare.

**Results:** Patient lack of comprehension regarding the test was the strongest predictor of non-adherence to follow-up. Older age, Arab ethnicity, and lower SES (socio-economic status) significantly reduced adherence. We found no correlation with gender, marital status, patient activation, waiting for time or distance from gastroenterology clinics.

Primary care physicians underestimate non-adherence rates. They feel responsible, but lack the time and skills to ensure adherence. Gastroenterologists do not consider FIT an effective tool for CRC detection. Lack of agreement between screening recommendations and gastroenterologist opinion and lack of awareness among healthcare authority figures negatively impact the screening program.

**Conclusions:** Interventions to improve follow-up after a positive FOBT should be targeted at all levels within the health care system. Individually tailored patient interventions that are educationally and culturally appropriate prior to testing completion is essential. Strategies to support primary care physicians in the test and follow-up process, as well as improving communication between physicians, and finally, increasing awareness among healthcare leaders will all improve outcomes.

**Health Policy Implications:** We have clearly defined avenues to improve colorectal cancer screening outcomes at all levels of the system.

#### Abstract 237 Partial smoking ban breaks the promise of smoke-free environment in Kazakhstan

##### Jamilya Sadykova^1^, Ardak Baizhaxynova^2^

###### ^1^National Coalition “Smoke free Kazakhstan”, Kazakhstan; ^2^Nazarbayev University, Kazakhstan

####### **Correspondence:** Jamilya Sadykova

**Background:** The fundamental human rights and principles of Article 8 of the WHO Framework Convention on Tobacco Control (FCTC) requires 100% smoke-free public places. Kazakhstan ratified the FCTC in 2006 but only has a partial smoke free policy; current law allows designated smoking rooms (DSR) in public dining establishments, while other public places must be 100% smoke free.

**Study Question:** Assess the effectiveness of specially designated places for smokers, in protecting people from second-hand smoking exposure, in the public dining venues of Kazakhstan by means of air quality monitoring.

**Methods:** A cross-sectional study of indoor air quality was conducted from September to October 2017 in the largest city of Kazakhstan. A total of 29 public dining establishments with different smoking policy were monitored in the evenings. The real-time measurement of PM2.5 particulate matters was conducted by TSI SidePak AM510 Personal Aerosol Monitor and was ranked using the WHO target air quality guideline.

**Results:** The highest mean PM2.5 level was detected inside the DSRs (648 μg/m3), followed by venues with similar results where smoking was allowed throughout the venue (180,3 μg/m3) and inside the smoking hall (182 μg/m3). The third rank belongs to non-smoking areas venues which allows smoking only in DSRs (73.1 μg/m3). The lowest mean PM2.5 level was observed in 100% smoke-free venues (26 μg/m3).

**Conclusions:** PM2.5 concentrations at DRSs which comprise hazardous level severely undermines the smoke-free environment national agenda. Unhealthy levels of PM2.5 concentrations at smoking areas confirm that a partial smoking ban violates the fundamental human right to be protected from second-hand smoke.

**Health Policy Implications:** Air quality monitoring data confirms that anything less than 100% smoke-free policies is hazardous to people and must be amended to eliminate DRSs and other exemptions from public places, as mandated by the Framework Convention on Tobacco Control.

### Parallel 2

#### Abstract 214 Using quality data to strengthen routine immunization in Nigeria; experience with routine immunization (RI) lots quality assurance sampling (LQAS)

##### Rilwan Raji

###### World Health Organization, Nigeria

**Background:** Studies emphasize the importance of quality data for effective planning, monitoring, and evaluation. The 2015 National Immunization Coverage Survey (NICS) in Nigeria revealed data quality issues across all 36 states, (worse in 18 states) of the Country with National Penta3 Coverage at 33% as against Administrative (Admin) Coverage of 98%, hence the importance of RI LQAS to identify reasons for disparities.

**Study Question:** Does the RI data from health facilities (HFs), aggregated at districts (LGAs) reflect true performance? What reasons explain disparities? What are the primary sources of information that guide vaccination amongst caregivers?

**Methods:** 18 priority States were selected. Using a probability proportionate to population size, all LGAs in a State, 6 HFs and 6 settlements were selected. Within each settlement, 10 households (HH) were selected from which 1 child 0-11 months per HH was selected for assessment by reviewing the immunization card and/or mother’s recall. Reasons for partially/not immunized for age and sources of information about vaccination were ascertained.

Using cumulative binomial probabilities, a district (Lot) was said to have passed (accepted at 80% coverage or more) if at least 8 of 60 children sampled were fully immunized for age.

**Results:** A total of 2,292 sites, 22,920 households, 22,920 children 0-11months sampled in 382 districts. Only 3% of the districts passed, with 56% of the States having < 80% coverage. At the individual level, only 1 of 3 children in the Country was fully immunized for age.

The main reasons for none/incomplete immunization was weak demand for immunization with Health Workers (HWs) being the major source of information about vaccination in the Country.

**Conclusions:** The RI LQAS confirmed findings of the NICS that admin data did not reflect RI performance in Nigeria. Subsequently, a State of Emergency on RI with RI Coordinating Centers set up across States with RI LQAS conducted quarterly to review performance.

**Health Policy Implications:** Instituting frequent low budget surveys as RI LQAS is effective in ensuring reliable data for planning to improve vaccination of children and enshrine accountability amongst erring HWs that falsify data.

#### Abstract 103 First year of the first population-based biobank in Israel

##### Daniella Beller

###### Maccabi Institute for Research and Innovation, IL

**Background:** Maccabi Healthcare Services (MHS) has set for itself the target of creating the first population-based biobank in Israel, thus providing an ideal platform for Precision Medicine research. MHS is the second largest healthcare provider in Israel. MHS serves 2.3 million members which constitutes a representative quarter of the Israeli population. Electronic health records have been implemented in MHS for over a quarter of a century. With less than 2% annual member turnover, MHS records have a longitudinal history of many patients throughout their entire lives. In 2017 the Tipa Biobank (Tipa) was launched, a nationwide biobanking program to link samples and EHR data for broad research use.

**Study Question:** We describe the initial phases of implementation of a large-scale population-based biobank initiative within the setting of a public healthcare fund.

**Methods:** Patients are approached by trained concenters and offered to participate. Samples are collected in a standardized manner and transported daily using the existing nationwide infrastructure of MHS.

**Results:** As of March 2019, over 70,000 MHS patients consented to participate in Tipa and over 100,000 vials have been stored. Of the patients approached, over 50% consent to participate. Tipa participants are slightly older (mean age of 49.7) and are more likely to be female (60%). 23% of participants are diagnosed with hypertension and 18% of them suffer from diabetes of pre-diabetes.

**Conclusions:** With virtually no funding MHS succeeded in creating the first population-based biobank in Israel. Tipa is a resource that enables a new model for translational research that is faster, more flexible, and more cost effective than traditional clinical research approaches. The model is scalable, and will increase in value as resources grow.

**Health Policy Implications:** Findings from using this resource will impact the development of personalized medicine tools and which will not only impact Tipa participants but will change the way patients are treated.

#### Abstract 276 Innovative adoption of technology for improving population based screening of common non communicable diseases in India

##### Aman Kumar Singh^1^, Rajeev Kumar^2^, Manas Pratim Roy^2^, HSD Srinivas^3^, Sita Ram Budaraju^3^

###### ^1^Tata Trusts; Ministry of Health and Family Welfare, India; ^2^Ministry of Health and Family Welfare; Government of India, India; ^3^Tata Trusts, India

####### **Correspondence:** Aman Kumar Singh

**Background:** India is witnessing a rapid health transition with a rising burden of Non-Communicable Diseases (NCDs) which account for around 5.8 million or 61% of all deaths annually.

The Government of India has launched the world’s largest government-funded health Programme “Ayushman Bharat” to strengthen comprehensive primary health care, reduce out of pocket expenditure and conduct Population-Based Screening (PBS) for Diabetes, Hypertension & Common Cancers (oral, breast and cervical) for population aged>30 years; targeting 500 million population annually.

**Study Question:** Does adoption of Technology hasten and standardize prevention, control and management of Non-Communicable Diseases?

**Methods:** A NCD application software has been developed and deployed by Tata Trusts and Dell (as development partner) for the Government of India to facilitate PBS of common NCDs. It automates intensive screening, digitalizes health records, standardize recording and reporting system and helps in NCD management through decision support system (DSS), streamline upward and downward referrals across health facilities, tracking follow up and preparation of work plan for the health personnel.

**Results:** Tata Trusts is conducting an intensive capacity building of front-line workers (FLWs) for technology adoption. Till date, 11,450 personnel have been trained who have enrolled 45.6 million persons in NCD application of which 29.2 million persons have been screened for common NCDs from September 2018-March 2019.

**Conclusions:** With strong political will, regular capacity building and supportive supervision it is possible to scale up adoption of technology by FLWs enabling screening of large populations for common NCDs within a short span of time.

**Health Policy Implications:** The Government of India has adopted the use of NCD application as one of the basic digital tools for record keeping and NCD management.

#### Abstract 34 Big data as a catalyst for policy research, pharmaco-epidemiology and cluster randomized clinical trials

##### Vincent Mor

###### Brown University, USA

**Background:** Interventions requiring a restructuring of health care systems are rarely replicated and difficult to extend beyond select early adopting health care organizations. However, since most program innovations require adoption by functioning health care systems, the classic NIH research phase models ignore this crucial implementation and dissemination phase which also needs to be rigorously tested.

**Study Question:** What are the necessary conditions under which a health systems intervention can be successfully implemented in the real world of functioning health care systems?

**Methods:** We examined multiple pragmatic, cluster randomized clinical trials implemented in functioning health care systems in order to identify the characteristics of interventions that are associated with successful replication and implementation of the intervention. A total of 5 different types of interventions were examined with successively more complicated interventions.

**Results:** Interventions that merely substitute one treatment for another are readily implemented with very successful adherence to the intended design. As interventions require changes in assignments, allocation of resources and shifts in expectations of supervisors and staff, the degree of adherence to the research model of the intervention breaks down considerably. The more the breakdown in intervention adherence, the greater the effect of the intervention must be in order for it to demonstrate the intended effect on patients’ outcomes.

**Conclusions:** Understanding the potential that innovations in health care practice can achieve the intended effects requires careful consideration of the level of organizational disruption that each entail since health care organizations have considerable entropy which can reduce the likelihood that the desired outcomes are achieved.

**Health Policy Implications:** Before interventions, shown to be effective in leading organizations, are widely promulgated across the country, it is essential that researchers and policymakers understand how the necessary organizational changes in daily practice can be achieved in the average health care organization.

#### Abstract 116 Towards a national system for measuring and public reporting of waiting time for community-based specialist care

##### Rachel Wilf Miron^1^, Ilya Novikov^2^, Arnona Ziv^2^, Avishai Mandelbaum^3^, Ya’acov Ritov^4^, Zach Tagar^1^, Osnat Luxenburg^1^

###### ^1^Ministry of Health, IL; ^2^The Gertner Institute for Epidemiology and Health Policy Research, IL; ^3^Technion, Haifa, IL; ^4^University of Michigan, USA Information Systems, USA

####### **Correspondence:** Rachel Wilf Miron

**Background:** Extended waiting time for a physician's appointment may incur health, financial and social consequences. The Israeli Ministries of Health and Finance initiated a National Program for Reducing Waiting Times (WT). However, attempts to develop national indices for WT have encountered challenges, mainly due to differing measurement approaches and infrastructures among the Israeli healthcare providers.

**Study Question:** Develop a unified national methodology to measure WT for community-based specialist care and to create the appropriate format to present it to the public.

**Methods:** Existing infrastructures of health maintenance organizations (HMO) were analyzed, in search of a “common denominator” methodology. Measures were applied to the five most common medical specialties. An interactive application, based on geographic information systems, was developed for public reporting of WT.

**Results:** We developed an algorithm to calculate WT that HMOs offer their members, by geographic regions, in two formats: “any physician” and “specific physician”. Supply estimation was based on 2 million available appointments, collected during October and December 2018. A clinic's activity served as an estimator of demand for its physician. The interactive application presents various statistical measures for districts and towns, by medical specialty. WT distribution differed among specialties. WT in central Israel did not always present shorter WT when compared with geographic peripheries.

**Conclusions:** The first stage of developing the national system for measuring WT was completed through cooperation with stakeholders and will serve as a platform for continuous dialogue. Future stages entail the development of an information infrastructure to enable measurements of actual WT, accompanied by a comprehensive survey towards understanding patients’ journeys when making appointments.

**Health Policy Implications:** Despite diverse challenges, national reporting of WT will be accessible to the public in April 2019, encouraging transparency and evidence-based discourse among decision-makers. Identification of barriers will serve to allocate resources, improve WT and strengthen public health services.

#### Abstract 167 Home sweet home

##### Yael Applbaum, Ziona Haklai, Ethel-Sherry Gordon

###### Ministry of Health, IL

####### **Correspondence:** Yael Applbaum

**Background:** One of the Israeli health system's sorest points is the overcrowding of general medicine departments.

A society that is living longer with more chronic illnesses and a shortage of chronic care beds, have caused the acute care medicine departments to bear a great part of this burden.

The occupancy rates in medicine departments are 97-107% with seasonal changes. Seeing patients lying in a bed in the hallway of a medical ward is commonplace.

Many suggestions have been raised to alleviate the problem. One recommendation was to develop a stronger system of home hospitalization.

**Study Question:** If indeed home hospitalization could shorten the stay of some patients, how much of the burden would decrease?

**Methods:** We chose three diseases that are amenable to continue treatment at home after the initial evaluation and inpatient treatment: congestive heart failure, pneumonia and urinary tract infections. We measured the number of discharges and the average length of stay of patients with these diseases listed as the primary diagnosis and estimated how many beds we could save if we could decrease the length of stay for patients with these three diseases. All the data were collected from the national hospitalization database in the Israel Health Ministry.

**Results:** There were 32,000 discharges for these three diseases in 2016, with the average length of stay between 5-5.7 days. Assuming we had an alternative setup such as home care that would allow us to decrease the average length of stay by two days, we estimated that we would save 60000 bed days, reducing the occupancy by 5%, or, alternatively, reduce the number of beds by 165.

**Conclusions and Health Policy Implications:** Although this is an exercise based on very crude but large data, in this very conservative model, we show that establishing an intensive home-based care system that would allow reducing the length of stay could help decrease the burden of hospitalization in the general medicine wards.

#### Abstract 288 Health service integrated construction in agricultural & pastoral villages and townships of Qinghai, China

##### Yongnian Liu, Rong Wang, Mingyu Huang, Xiang Gao

###### Medical College, Qinghai University, China

####### **Correspondence:** Yongnian Liu

**Background:** Qinghai Province is located on the Tibet Plateau, covering a large area (722,300 km^2^), but with a small population (5.73 million). People living in the remote have poor access to health services.

**Study Question:** To describe the current existing integrated management of rural health services, and to analyze the determinants of the quality of rural health service, thus improving the health service capacity in Qinghai, particularly among the rural minority villages and townships.

**Methods:** We have selected the pilot areas through stratified sampling from the aspects of health services utilization and satisfaction, performance and changes in the health sector, and conducted surveys on rural health services and health authorities in the pilot areas and surveys on agricultural and pastoral households residents, and qualitative interviews with medical staff and medical managers to understand the implementation and problems on integration management and improvement of rural health service.

**Results:** With the achievements of the project, the village doctors have obtained the identities equal to that of social welfare jobs, e.g. pension and medical insurance. We have put forward multi-compensation system on village doctor post subsidies, zero added profit drug subsidy, water, electricity and heating subsidies running costs, network operating subsidies. Besides, we have established systems on conducting business guidance in medical institutions of villages, townships and counties, technical support, personnel training and training system.

**Conclusions:** The central pharmacy has been established for village clinics to be engaged in unified order, drug distribution and supervision on drug utilization. The minority clinics have been established, which are the indispensable in rural health service.

**Health Policy Implications:** The achievements on the resources being integrated, the management being accessed and service quality and capacity being promoted could be realized to build an appropriate model for rural health service in Qinghai.

#### Abstract 286 PRO (Patient Reported Outcomes) implementation at Sheba Medical Center

##### Alex Galper, Ora Shamai-Rosler, Varda Stanger, Eyal Zimlichman

###### Sheba Academic Medical Center, IL

####### **Correspondence:** Alex Galper

**Background:** Patient-reported outcomes measure patients’ views of their health-related quality of life as received directly from the patient. With the increasing interest and usage of PRO's in the world, Sheba Medical Center is the pioneer in the field in Israel, since 2015, looking at the potential value in PRO implementation in routine practice as a quality improvement and decision support tool together with the opportunity to drive changes in healthcare delivery.

**Study Question:** Feasibility of PRO collection and reporting platform at Sheba Academic Medical Center.

**Methods:** A comprehensive process, initiated by department leading clinician and involving multidisciplinary staff, results in a tool set build up and department implementation. The tool set used to gather the PRO data from patients in various methods, on specific periods, processed and presented in an easy to read format in the EMR.

**Results:** Program continues growth from three to over fifty areas in various implementation stages doubled the number of questionnaires collected and patients enrolled between 2018 (5,784 questionnaires, 3,475 patients) and 2017 (2,542, 1,815) and more than tripled in relation to 2016 (1,729, 479).

The increased use of the patient portal had a significant impact and involved 11% of all complete tool sets and 30% of the follow-up questionnaires.

**Conclusions:** Program expansion shows success in increasing number of patients and fields enrolled. In order to scale up to include large target populations, a better use of electronic mass data collection methods is required.

**Health Policy Implications:** Basic usage of a patient generated report in a clinical practice during patient-physician encounter provides insights on the ongoing patient journey that can help focus all parties on the patient status, goals and progress.

Combining patient centered care with data driven care and other EMR existing data can be used to create and measure outcome benchmarking as well as construct patient predictive tools to make informative treatment decisions and to perform as a clinical decision support tool for both patients and clinicians.

#### Abstract 268 Withdrawn

#### Abstract 140 Planning for specialists: can we predict the number of new specialists?

##### Myriam Aburbeh, Haim Karger, Ziona Haklai

###### Ministry of Health, IL

####### **Correspondence:** Myriam Aburbeh

**Background:** In recent years the proportion of specialist physicians in Israel has increased. Planning the medical workforce must consider cost and time of training new specialists.

**Study Question:** Can we predict the number of future specialists following their initial physician license by specialty?

**Methods:** Data was obtained from the licensed physician and residents’ databases. A cohort of physicians licensed in 2005-2008 was followed up to find time interval till the beginning of the first specialization, and till its completion, allowing a 10-year follow up. For those who did not complete the first specialization, we checked whether another specialization was begun.

**Results:** Of 2,351 physicians who received their license in 2005-2008, 1,964 began the first specialization, on average 13 months after receiving their license, with a median time of 5-6 months.

Following up these physicians shows that 27% did not finish their specialist training within 10 years from starting. Of those, 28% of internal medicine interns did not finish, 25% in psychiatry, 56% in general surgery, 49% in anesthetics, 18% in family medicine and gynecology and obstetrics and 8% in pediatrics.

From those who did finish their first specialty, 81% of internal medicine interns received their specialist license within 6 years, 84% in family medicine and pediatrics, and 74% in psychiatry. 78% of those in general surgery finished in 8 years, and 91% in gynecology and obstetrics.

In most specialties, median time till finishing was longer for male than female physicians.

A third of those who did not finish their first specialization began and finished a different one; 45% of those who didn't complete family medicine and pediatrics, 38% - gynecology and obstetrics and 52% - general surgery.

**Conclusions:** A follow up of medical specializations from initial license to completion allows mapping their supply and demand.

**Health Policy Implications:** Planning the medical workforce for changing population needs should utilize medical resident data.

## B: “Uber’ization” of healthcare - dream or nightmare? –

### Abstract 210 Measuring the quality of a therapeutic meeting using objective-structured clinical simulation (OSCS) - the case of diabetes educators

#### Orly Tamir^1^, Tatiana Kolobov^1^, Vardit Kalamaro^1^, Karen Hershkop^2^

##### ^1^The Gertner Institute for Epidemiology and Health Policy Research, IL; ^2^Shaarei Tzedek Medical Center, IL

###### **Correspondence:** Orly Tamir

**Background:** Under a national endeavor to establish the role of Diabetes Educators (DE) in the healthcare system, during 2015-2018 over 200 healthcare professionals were trained under the auspice of the Ministry-of-Health to serve as educators. The training comprised of theoretical knowledge and hands-on practice, with an internationally-unique accreditation exam of OSCS carried out by standardized patients in clinic-like settings. Up until now, evaluation of the simulated therapeutic meetings was conducted by trained evaluators, but in a subjective manner. In order to assess the suitability of the training to the DE role and establish the accreditation system, there is a need for a reliable and valid quantitative evaluation.

**Study Question:** (1) To examine the use of OSCS to modulate a real-life therapeutic-meeting. ((2 To develop and validate an instrument for quantitative evaluation of the OSCS.

**Methods:** All DE were asked to provide feedback through a self-report questionnaire on the suitability of the OSCS modality. In addition, an evaluation tool for the accreditation exam was constructed on the basis of well-established relevant scales and consultation with experts. The validity and reliability of the instrument were tested by several independent rounds of evaluations, having corrections to the inventory after each round. Cost components of OSCS per participant were collected.

**Results:** Over 90% of the participants expressed satisfaction from the compatibility of OSCS with their everyday work and recommended to continue with this method. The main parts of the final version of the instrument include: general impression, the structure of the meeting, obligatory communication skills, deeper level skills and summary score. The validity and reliability scores of the OSCE evaluation were high. The cost per participant was calculated to be USD 150.

**Conclusions:** OSCS is a suitable methodology for testing the quality of a therapeutic meeting in a clinical setting. The suggested instrument was proved to allow credible evaluation.

**Health Policy Implications:** Implement the evaluation tool for accreditation tool as well as for the routine assessment of DE in practice.

### Abstract 216 A web-based discussion for difficult-to-solve cases: a promising experience in a large physician network

#### Shmuel Prints^1^, Tsernishov N.A.^2^

##### ^1^Neglected Diseases Collaboration, IL; ^2^Doctor na rabote (DnR), Russia

###### **Correspondence:** Shmuel Prints

**Background:** A diagnosing of rare diseases takes an unusually long time. During that period, the patient gets 2-3 misdiagnoses. Educational web presentations of patients with rare diseases show that among the participants, at least one has the right answer. An off-line discussion about a patient with an unusual clinical picture may be a promising opportunity for a physician experiencing diagnosing difficulties.

**Study Question:** We studied the number of diagnostic assumptions and the speed of their receipt in the web-based consultation regarding patients who standard diagnostic procedures did not allow to establish a diagnosis**.**

**Methods:** The social network “Doctor na rabote” (DnR) brings together more than half a million Russian-speaking physicians. Almost every day, its members turn to their colleagues with questions about diagnostic difficulties. We conducted an observational study of difficult-to-solve cases, presented on DnR in the period 1.09.18 - 02.28.19. In order to analyze the practice of a web Concilium regarding patients with possible rare diseases, we chose cases in which at least 20 doctors participated. We investigated the time between publication and the receipt of the last diagnostic assumption, the number of independent diagnostic assumptions and the total number of participants in the discussion.

**Results:** During the study period, there were 25 discussions that met the inclusion criteria. Two cases of them were presented twice. The average number of participants who made significant comments was 27.32 (20-41). The count of independent diagnostic assumptions per case varied from 6 to 22 and averaged 12.6. The time between publication and receipt of the last of the diagnostic version did not exceed two weeks.

**Conclusions:** An off-line Concilium regarding difficult-to-diagnose cases yields a significant number of diagnostic assumptions in a very short time.

**Health Policy Implications:** An adding to off-line web Concilium a tool for choosing between assumptions can significantly speed up the diagnosis of such patients in everyday medical practice.

### Abstract 38 Personal and social patterns predict influenza vaccination decision

#### Dan Yamin^1^, Adir Shaham^1^, Gabriel Chodick^2^, Varda Shalev^2^

##### ^1^Tel Aviv University, IL; ^2^Tel Aviv University; Maccabi Healthcare Services, IL

###### **Correspondence:** Dan Yamin

**Background:** Seasonal influenza vaccination coverage remains suboptimal in most developed countries, despite longstanding recommendations of public health organizations. The individual’s decision regarding vaccination is located at the core of non-adherence.

**Study Question:** Can we predict whether a patient will become vaccinated against influenza in the next season?

**Methods:** A retrospective longitudinal cohort study, utilizing data from the electronic medical records of 250,000 members of Maccabi Healthcare Services.

The data was collected from 1,600 clinics, 32 hospitals and 700 pharmacies, between the years 2007 and 2017.

We developed machine-learning models to predict the future vaccination decision of a patient. Models’ performance was based on the area under the ROC curve.

**Results:** The vaccination decision of an individual can be explained in two dimensions – personal and social. The personal dimension is strongly shaped by a “default” behavior, such as vaccination timing in previous seasons and general health consumption, but can also be affected by temporal factors such as respiratory illness in the prior year.

In the social dimension, a patient is more likely to become vaccinated in a given season if at least one member of his family also became vaccinated in the same season (RR:11.09; 95% CI:10.92 to 11.25). Furthermore, vaccination uptake was highly associated with the individual’s home geographic area (P value<.0001), and with the individual’s socioeconomic score. An XGBoost-based predictive model achieved a ROC AUC score of 0.91 with accuracy and recall rates of 90% on the test set. Prediction relied mainly on the patient’s individual and household vaccination status in the past, age, number of encounters with the healthcare system, number of prescribed medications, and indicators of chronic illnesses. A model which relies solely on the vaccination timing in the previous season yielded 0.81 ROC-AUC scores.

**Conclusions:** The decision of an individual to become vaccinated is highly predictable.

**Health Policy Implications:** Our study sets a major step toward personalized influenza vaccination campaigns.

### Abstract 243 Automatic evaluation of routine computed tomography scans for prediction of osteoporotic fractures

#### Noa Dagan^1^, Eldad Elnekave^2^, Noam Barda^1^, Eitan Bachmat ^3^, Ran Balicer^1^

##### ^1^Clalit Research Institute; ^2^Zebra Medical Vision LTD., IL; ^3^Ben-Gurion University of the Negev, IL

###### **Correspondence:** Noa Dagan

**Background:** Osteoporotic (OP) fractures cause major morbidity and mortality. The clinical importance of fracture risk prediction models such as FRAX is well established, but these models require many inputs and are underutilized in practice.

**Study Question:** How does the predictive ability of OP-fracture risk scores, derived automatically from routine CTs using an artificial-intelligence based algorithm, compared to that of FRAX?

**Methods:** Members of Clalit Health Services aged 50-90, who underwent routine chest or abdomen CTs prior to July 2012, were included.

An algorithm scored each CT for bone mineral density and the presence of vertebral compression fractures. Three models predicting five-year fracture risk were assessed as of July 2012: FRAX, CT (using algorithm markers together with CT metadata of age and sex), and combined FRAX-CT. The two outcomes were major OP and hip fracture incidence over 5-years (2012-2017).

Model discrimination was evaluated using the area under the ROC curve (AUC). Missing FRAX inputs were multiply imputed. Significance was evaluated using 500 bootstrap samples.

**Results:** A total of 48,227 individuals were analyzed. Of these, 5,106 (10.6%) and 1,901 (3.9%) suffered major OP and hip fractures during follow-up, respectively.

The AUCs of the FRAX, CT and combined models were 69.1%, 71.0% and 72.3%, respectively for major OP fractures, and 75.1%, 76.0% and 77.2% for hip fractures, respectively. All AUC results were significantly different except those between the FRAX and CT models for hip fractures.

**Conclusions:** Fully automatic screening for OP fracture risk using routinely acquired CT scans achieves discrimination which is at least as good as the well-established FRAX model. If data for all required inputs is available, combining the CT scores with FRAX further improves predictive ability.

**Health Policy Implications:** OP fracture prediction scores can be added automatically to CT reports, to help identify people at risk for fractures who are currently missed.

### Elevator Pitch

#### Abstract 206 Patient navigation in a virtual world: using english speaking immigrants as a model for telehealth advocacy

##### Aviva Yoselis, Gabriel Pransky

###### The Shira Pransky Project, IL

####### **Correspondence:** Aviva Yoselis

**Background:** Although there are no exact figures, there are approximately 300,000 Native English speakers living in Israel, and 5,000 more making Aliyah every year. Despite the demand of the Knesset to make healthcare accessible, both culturally and linguistically, to immigrants, English speaking services have been slow to come. This is due, in part, to the belief that all healthcare providers can converse in English (they cannot), and that English speakers making Aliyah navigate the healthcare system effectively (they do not). These discrepancies cause (1) decreased access to needed healthcare services, (2) increased levels of frustration, disempowerment and depression (3) decreased quality of life among English speaking immigrants dealing with healthcare issues. In addition, this immigrant population needs to overcome the cultural stigma of entitlement and the true lack of social capital that healthcare navigation operates through Israel.

**Study Question:** Can effective navigation and advocacy services be delivered to a varied and dispersed immigrant population, virtually, and have similar, if not better, outcomes than standard in-person services offered by the Ministry of Welfare, National Insurance Institute and the Ministry of Health?

**Methods:** An online system was developed that delivers health navigation and advocacy services based solely on online and ongoing telephone service, in conjunction with the information database of “Kol Zchut”, All Rights Organization.

**Results:** Over a two-year period, over 2,000 English speakers have been served by this online system, and over 100,000 individuals have received accurate healthcare information in English through the collaboration between The Shira Pransky Project and the All Rights site. More importantly, over 400 immigrants in the periphery of the country, who have much less access to English speaking services have received services.

**Conclusions:** An organized informational and online-based system, with trained staff can deliver effective informational and advocacy services. A culturally diverse population spread out over a large geographic area can receive effective intervention services that can mitigate crises, increase access to benefits and suitable care.

**Health Policy Implications:** A system that generates empathy even if the platform is Telehealth (i.e. not in person), can be personal without being in person. Using trained staff and a specific rubric of service delivery and intake, services can be delivered effectively, efficiently and with minimal costs.

#### Abstract 47 Defining cultural factors in the subjective burden experience, amongst Arabic Muslim mothers to a son/daughter with a severe mental disability

##### Muhammad Igbarya

###### Ministry of Labor, Social Affairs and Social Services, IL

**Background:** The act of diagnosing a mental illness amongst one of the children in the family changes the family balance, burdens it with a heavy stigma and makes parental care more difficult. The influence of the disease on the family and parents was researched in relation to the subjective burden concept, a burden which describes the negative psychological consequences of the disease on those treating it as feelings of loss, depression, fright and embarrassment.

**Study Question:** The research examines how Arabic, Islamic mothers describe and experience the subjective burden that is cast upon them in light of treating the harsh mental disability of their children. Furthermore, it examines the cultural and influences of cultural motives in the construction of the motherly burden experience

**Methods:** The research included 30 married Arabic mothers who live in the state of Israel. They were part of a ‘purposeful’ sample. The research interview included 19 qualitative questions, in addition to a socio-demographic questioner containing 16 questions. The findings analysis was performed according to the qualitative methodology, and according to the Grounded Theory of Strauss and Corbin.

**Results:** The research found six areas in that affected the motherly burden: the religious factor; the types of motherly emotions; the perception of the illness; contact with professionals’ stigma’ and changes in the Arabic family. Socio-demographic factors that are unique to the Arabic family such as: low socio-economic status, mother's education, high number of children, lack of gender equality, low accessibility to social services, and families with multi-generational distress, all put an even greater motherly burden.

**Conclusions:** Mothers of daughters reported a subjective load and burden that were higher than mothers to boys with mental disabilities. A heavy subjective burden was reported by mothers of families that are suffering from multi-generational distress, such as: unemployment; poverty; sickness; drug dealing; and crime.

**Health Policy Implications:** These findings helped the understanding of the unique motherly needs, and thus helped develop specific intervention programs focused at giving an answer to the consequences of the motherly burden, in addition to helping form a macro level policy to the Arab society in Israel.

#### Abstract 265 Maccabi red - uberization of minor trauma and semi urgent medical care in primary care setting

##### Ilan Yehoshua, Limor Boker Faran, Neomi Siegal, Miri Reuveni Mizrachi

###### Maccabi Healthcare Services, IL

####### **Correspondence:** Ilan Yehoshua

**Background:** Minor trauma and semi-urgent conditions are a burden on the health system. In a year, in Maccabi alone, there are more than 50,000 cases. Many patients that seek urgent medical care are treated in the emergency department. The Maccabi health service has the medical personnel which is capable of delivering the same, if not better treatment, in the outpatient setting. This treatment is faster and cheaper. In order to facilitate the needs of the individual patient, we need a system that will connect the patients to the Doctors enrolled to program within the relevant area efficiently. It is very similar to the Uber system. We call this medical service innovation ‘Maccabi red’. For example - a 3 years old child with a laceration who needs suturing; the ‘Maccabi red’ system can efficiently find the Doctor that can perform the suture. Thanks to this system the child and his family do not need to go to a busy emergency room thus reducing the burden of disease and health costs.

**Study Question:** (1) How efficient is this system in the management of minor trauma and semi-urgent conditions? (2) How does the project influence the satisfaction of Doctors and patients? (3) What are the changes in cost and health burden?

**Methods:** Since 12/2017, a pilot of 219 Doctors from different specialties and selected regions around the state was trained and enrolled. Each condition is referred to the system and directed accordingly to the suitable Doctor close by.

Ongoing evaluation of the above questions is being performed.

**Results:** (1) Until February 2019, 2,065 referrals were made to the Maccabi Red. 78% were suitable for the definitions of the program. (2) Patients and Doctors’ satisfaction were very high in qualitative questioners. (3) Financial benefit – still not enough data.

**Conclusions:** This is a useful process that needs ongoing adjustments to become a model for better utilization of medical resources**.**

**Health Policy Implications:** Maccabi Red could serve as a good example of how can ‘Uberization’ of the medical system succeed.

#### Abstract 166 Clinical trials in advanced therapies - Israel is on the map

##### Shlomo Yaacobi, Miriam Cohen-Kandly, Catherine Ela, Eli Marom

###### Ministry of Health; IL

####### **Correspondence:** Shlomo Yaacobi

**Background:** Advanced therapies are breakthrough technologies in the medical world designed to treat unmet medical needs including cancer, neurodegenerative and hereditary diseases.

Nationally there is great medically and financially importance, in the development and implementation of innovative treatments.

**Study Question:** Does the Ministry of Health's policy enables developments in advanced therapies in Israel?

**Methods:** Due to the progress and complexity of these innovative experimental treatments, the MoH nominated specific committees to address necessary aspects, such as clinical safety data, scientific integrity, product quality and ethical value. In 1999, a central committee for clinical trials utilizing cells and tissues medicinal products was established, and in 2007 a committee for gene therapy clinical trials was established. In 2012, committee activity became regulated according to annual schemes.

In 2015, the Ministry of Health created a computerized database of all clinical trials. The database includes trial documents, product classification, MoH processing time, applications status, safety and annual reports, etc.

The Department of Clinical Trials conveys MoH policy throughout conferences and working groups and one on one meetings with sponsors and institutional review boards.

**Results:** Summarizing the data from 2015-2018, we can identify an increase in the number of innovative experiments (6 in 2015 to 22 in 2018) in all phases.

The processing time is usually between 150 to 200 days. Over the years 2015-2017 MoH approved over 70% of applications.

MoH also approved compassionate use of these treatments for patients that could not be included in a trail.

**Conclusions:** The Ministry of Health's policy enables and promotes developments in advanced therapies in Israel.

**Health Policy Implications:** In order to further promote these innovative trials, the MoH acts to improve the approval process, minimize evaluation time, enable transparency, whilst at the same time guarantee the safety of the participant in the trial.

#### Abstract 48 Clinical outcomes, QoL and costs analyses of telemedicine application in lower extremities ulcers treatment

##### Alexander Gamus, Varda Shalev, Gabriel Chodick

###### Maccabi Healthcare Services, IL

####### **Correspondence:** Alexander Gamus

**Background:** The estimated annual prevalence of the foot and leg ulcers n Israel, where the prevalence of diabetes mellites (DM) estimated at 8.4 %, up to 15% of DM patients may develop diabetic foot ulcers. Providing medical services to Lower Extremities Ulcers (LEU) patients in geographically remote regions is a growing concern in healthcare systems. Telemedicine (TM) has been suggested to be a potential solution to this problem.

**Study Question:** The study aimed at assessing the clinical effectiveness, cost-effectiveness, and quality of life (QoL) of TM application.

**Methods:** The research was conducted at Maccabi Healthcare Services, a 2.2 million-member sick fund in Israel, and performed during Jan 1^st^, 2013 – Jun 31^st^, 2017 period. Both TM and face-to-face modalities were implemented using identical treatment settings with the same nurse at each location. The same specialist supervised patients in each modality.

**Results:** A total of 650 cases (nTM=277, nFTF=373) with 5,203 visits. Comparable (P=0.475) proportions of healed ulcers (52% in TM vs. 55% in FTF) were detected. Survival analyses found a non-significant advantage of TM (0.887; 0.650-1.212) compared to FTF. A total of 83 TM and 94 FTF patients’ questionnaires included in the QoL trial. The mean quality-of-life in TM was 0.546(±0.249) compared to an FTF cohort with 0.507(±0.238), p=0.291. The cost-per-patient in TM, compared to FTF, was 7% higher; however, with similar quantities of TM and FTF patients, the cost in TM becomes lower. The alternative of FTF-only treatment demonstrated higher direct cost-per-patient by 30%.

**Conclusions:** Synchronous video-conferencing based TM may be a feasible and efficient method of LEU management.

**Health Policy Implications:** The Costs and Benefits study brings new evidence of cost per patient to a LEU treatment domain with little previous research.

### Parallel 2

#### Abstract 232 Fetal tele-monitor system – central analysis, quality control and archiving – preliminary account of the first year

##### Dror Raif^1^, Ofer Tadmor^2^, Yaakov Segal^2^, Miri Mizrahi-Reuveni^2^, Rachel Fish^2^, Angela Ironi^2^, Nachman Ash^2^

###### ^1^Hadassah-Hebrew University in Jerusalem, IL; ^2^Maccabi Healthcare Services, IL

####### **Correspondence:** Dror Raif

**Background:** Maccabi Healthcare Services provides healthcare services nationwide. One of those services is a prenatal evaluation of fetal well-being through a non stress test (NST). Maccabi's healthcare professionals perform 60,000 NST monitors per year in 142 care centers.

**Study Question:** What are the technical probability and the medical and economic benefit of fetal tele-monitor analysis and archiving via the Internet?

**Methods:** In 2017, the tele-monitor system dealt with 27,695 fetal monitors. Those consisted out of 2,697 monitors centrally analyzed by six doctors via a secure network, and 18,799 centrally archived post local analysis. The medical benefit of the project was studied and determined according to medical intervention and outcome of cases centrally analyzed.

**Results:** We found that 0.8% of real-time analyzed tele-monitors were diagnosed with fetal distress, and out of those, 78% were rushed to the hospital by ambulance and received a significant medical intervention, possibly altering the course of the pregnancy. Furthermore, 31.6% of archived monitors were inspected post archiving. Out of those, 0.42% were deemed incompatible to original analysis, and 37% of them received significant medical care.

**Conclusions:** The tele-monitor system was proven to improve the medical care of Macabbi outside large cities. The project proved that a limited staff can provide care in real time, with no geographic limitations. Moreover, the system contributed directly to the improvement of the medical care of some patients and enabled the inspection and monitoring of the quality of medical care of other patients.

**Health Policy Implications:** Secure internet networks are becoming increasingly widespread, allowing more services to be made available to more patients through tele-medicine. In light of the severe shortage of medical professionals and growing gaps in medical care between major metropolitan areas and more outlying areas, tele-medicine and care should be the next step in public healthcare.

#### Abstract 157 Does staffing levels of health-care workers impact retention in hiv care and treatment; a retrospective cohort analysis of data from Rift-Valley in Kenya?

##### Raphael Onyango

###### Kenya Medical Research Institute-Family AIDS Care and Education Services, Kenya

**Background:** Human resources for health is key to achieving the UNAIDS 90-90-90 targets by 2020. Retention in care is a step towards achieving viral load suppression among clients in HIV/AIDS care and treatment. The “Care&Treatment” program is implemented in 137 Ministry of Health facilities in the Rift Valley Province of Kenya.

**Study Question:** What is the impact of healthcare worker numbers on retention in care among People living with HIV (PLHIV)?

**Methods:** We performed retrospective analysis on routinely collected program data on staff and retention between October-December, 2018. These were staff numbers, adherence counselors, case managers/mentor mothers, nurses, and registered clinical officer (RCO). We calculated retention as a percentage of adults and children known to be on treatment 12 months after initiation of antiretroviral therapy and used Poisson Regression for count data controlling for facility-level clustering and using the total number of staffs at the facility level as an offset variable to assess the association between staff numbers and retention.

**Results:** There were 63/548(11.5%) -adherence counsellors, 296/548(54%)-case-managers, 97/548(17.7)-nurses, and 92/548(16.8%) registered clinical officers in 137 facilities. We observed statistically significant association between availability of overall staff numbers and retention, availability of 1-5 staff was IRR [0.35 95%CI (0.33-0.37)], 6-10 staff [IRR [0.14 95%CI (0.13-0.15)], and availability of above 10 staff numbers, IRR [0.055, 95% CI (0.052-0.060)]. Availability of adherence counsellor was protective [IRR 0.46 95%CI (0.44-48)]; 2-4 adherence counsellors [IRR 0.25 95%CI (0.23-0.27)]. availability of between 1-2 mentor-mother(s)/case-manager(s) was protective [IRR 0.40 95%CI (0.37-43)]; 3-4 mentor-mothers/case-managers [IRR 0.19 95%CI(0.18-20)]; above 5 mentor-mothers/case-managers [IRR 0.09 95% CI(0.08-0.10)]. availability of nurses was protective [IRR 0.56 95%CI (0.54-59)]. Availability of 1 RCO was protective [IRR 0.44 95%CI (0.42-46)]; between 2-4 RCOs [IRR 0.27 95%CI (0.25-0.29)].

**Conclusions:** Availability of healthcare workers improves retention and increased numbers improve retention in care among PLHIV.

**Health Policy Implications:** Understanding the distribution and allocation of healthcare workers will help the government to allocate scarce healthcare workers to achieve optimal health outcomes.

#### Abstract 4 Feasibility, safety, and effectiveness of a novel mobile application in cardiac rehabilitation

##### Robert Klempfner^1^, Amira Nachshon^1^, Irene Nabutovsky^2^, Liza Grosman^3^, Offer Amir^3^, Riki Tesler^4^, Yair Shapiro^4^

###### ^1^Sheba Medical Center, IL; ^2^Ariel University; Sheba Medical Center, IL; ^3^Baruch Padeh Medical Center, Poriya, IL; ^4^Ariel University, IL

####### **Correspondence:** Robert Klempfner

**Background:** Cardiac rehabilitation (CR) is underutilized globally despite evidence of clinical benefit. Major obstacles for wider adoption, include distance, travel-time and interference with the daily routine. Tele-rehabilitation can potentially address some of these limitations, enabling patients to exercise in their home environment or community.

**Study Question:** The aim of this study was to evaluate the clinical and physiological outcomes as well as adherence to tele-cardiac rehabilitation (tele-CR) in patients with low cardiovascular risk.

**Methods:** A total of 22 patients with established coronary artery disease participated in a 6-month tele-CR program. Datos Health, a novel digital health application and care-team dashboard were used for remote monitoring, communication and management of the patients. The primary objective of the study was to assess exercise capacity, determined by exercise stress test, using a treadmill before and following the 6-month intervention.

**Results:** Following the 6-month tele-CR intervention, there was a significant improvement in exercise capacity, assessed by estimated Metabolic Equivalents (METS) with an increase from 10.6±0.5 to 12.3±0.5 (P=0.002). High-density lipoproteins (HDL) levels significantly improved, whereas low-density lipoproteins (LDL), triglyceride (TG) glycosylated hemoglobin (HbA1c), systolic (SBP) and diastolic (DBP) blood pressure levels were not significantly changed. Exercise adherence was consistent among patients, with more than 63% of patents participated in a moderate intensity exercise program for 150 minutes per week.

**Conclusions:** Patients who participated in tele-CR adhere well to the exercise program and attained clinically significant functional improvement. Tele-CR program is a viable option for populations that cannot, or elect not to participate in center-based CR programs.

**Health Policy Implications:** Tele-CR is a viable option for attaining good adherence and functional improvement. Healthcare providers should strive to integrate alternative models of rehabilitation, such as Telehealth interventions tailored to individual’s risk factor profiles as well as community- or home-based programs to ensure there are choices available for patients that best fit their needs, risk factor profile, and preferences.

#### Abstract 176 New model of ‘Internet + Healthcare’: case study of online health services in China

##### Fowie Ng

###### Hong Kong College of Community Health Practitioners, Hong Kong

**Background:** China has one of the largest numbers of hospitals in the world. Access to good quality health care remains a problem in both urban cities as well as in the rural areas. Patients are rushing to outpatient clinics in well-known hospitals to register (guahao in Chinese) and have to wait to see the experts and specialists. The recent reform of the health services in China has facilitated the emerging giant online health services providers to fill in the gap in the form of ‘Internet + Healthcare’.

**Study Question:** (1) What are the current government policies and regulations in China towards the practice of ‘Internet + Healthcare’? (2) What are the current types of services and the model of this ‘Internet + Healthcare’? (3) Evaluate the current two major listed ‘Internet + Healthcare’ providers in the China market.

**Methods:** (1) Content analysis of the government policies and regulations regarding the development of ‘Internet + Healthcare’. (2) Case Study of the two major listed “Internet + Healthcare’ providers in China market in terms of their market, model and their outcomes.

**Results:** (1) A disruptive model of the health care market in China has been currently dominated by internet or financial giants. (2) Market share is constantly growing in this field. (3) Investment Funding and Initial Public Offering are the major sources of funding for sustaining and developing the services. (4) Changes in the behaviors of local citizens in seeking health advice and consultations.

**Conclusions:** While the current scale of ‘Internet + Healthcare’ provision in China is stilling developing, it is envisaged that there is a strong growing demand of adopting this new model to seek health services from the citizens in China. While these providers may not acquire their financial profits yet, this new model of economy has attracted much attention from the investors’ point of interest.

**Health Policy Implications:** (1) Can this model of ‘Internet + Healthcare’ be applied to other countries? (2) How to regulate and assure the quality of services or advices? (3) Urgent need for government to evaluate the effectiveness of the ‘Internet + Healthcare’. (4) Update and refine the current policy regarding ‘Internet + Healthcare’.

#### Abstract 269 Feasibility of telehealth in Honduras

##### Sandra Gomez Ventura^1^, Reyna M. Durón^1^, Dina Alvarez^2^, Andrea Summer^3^, Dean Sutphin^4^, Juan Pablo Bulnes^1^, Gabriela Mondragon^1^

###### ^1^Universidad Tecnológica Centroamericana, UNITEC, Tegucigalpa, Honduras; ^2^Hospital María de Especialidades Pediátricas, Tegucigalpa, Honduras; ^3^Medical University of South Carolina, Charleston, US; ^4^Edward Via College of Osteopathic Medicine, US

####### **Correspondence:** Sandra Gomez Ventura

**Background:** Telehealth is an innovative tool for teleconsultation and Tele-education. Our project unifies local and international efforts to establish the feasibility of this tool in Honduras. We pursue the benefit of patients and health personnel (especially medical students) in remote areas where the internet is available.

**Study Question:** Is Telehealth feasible in Honduras?

**Methods:** Our Interventional Pilot Project includes eight Telehealth stations in four Departments of the country: Two stations at Universidad Tecnológica Centroamericana’s (UNITEC) Campus, one at the public children’s hospital in the capital, one at a regional hospital (Danlí), and five at non-governmental clinics in Tegucigalpa, Copán and Olancho. The project follows international standards to protect privacy and confidentiality, as the standards by the American Telemedicine Association. We use three systems: Zoom for teleconferencing with Edward Via College of Osteopathic Medicine (VCOM), Vidyo for teleconferencing and teleconsults with Medical University of South Carolina (MUSC), and Aliv.io for teleconsult with the electronic record.

**Results:** Since 2017, UNITEC’s students connect with MUSC for conferences and case discussions, in 2019 they happen every week. Since 2018 they connect with VCOM to discuss cases related to public health strategies. Teleconsultations are developed per calendar. Physicians at the rural areas consult with specialists in pediatrics, physical therapy, neurology and internal medicine. Satisfaction rates are high due to financial savings and improved diagnosis and treatment plans, but there are complains related to internet connectivity and availability of more specialists for consultations.

**Conclusions:** This project has shown the feasibility of Telehealth in Honduras and could be a model to expand in the country to improve access to health care and continued medical education.

**Health Policy Implications:** This project includes education, diagnosis, electronic record and soon Tele-Diagnosis for radiology. For this reason, it is considered by the Honduran Government as a pilot that could provide inputs for the preparation of national Telehealth legislation.

#### Abstract 119 Digital patient portals and health outcomes, system efficiency and patient attitudes: a systematic review

##### Leonardo Villani, Andrea Barbara, Elettra Carini, Andrea Gentili, Angelo Maria Pezzullo, Walter Ricciardi

###### Università Cattolica del Sacro Cuore, Roma, Italy

####### **Correspondence:** Leonardo Villani

**Background:** Systems based on Electronic Health Records (EHRs) have the potential to improve patient care, self-management and activation. Among these systems, patient portals are becoming increasingly popular worldwide even though their impact on individual health and health systems efficiency is still unclear.

**Study Question:** The aim of the study was to analyze the impact of digital interactions between health systems or providers and citizens through patient portals or other EHRs-based systems on health outcomes, treatment adherence, health system efficiency, and patient characteristics, attitudes and satisfaction.

**Methods:** MEDLINE and Web of Science databases were queried from 1 January 2013 to 8 January 2019. Hypothesis-testing or quantitative studies of patient portals or other EHR-based digital applications that addressed patient outcomes, satisfaction, adherence, efficiency, utilization, attitudes, and patient characteristics, as well as qualitative studies of barriers or facilitators, were included.

**Results:** Forty-six articles were included: 2 randomized controlled trials, 19 observational hypothesis-testing studies, 14 quantitative descriptive studies and 11 qualitative studies. There is mixed evidence about the effect of portals on health outcomes, although most studies report positive results. Satisfaction is generally good, especially when the patients are involved in portal framing. The effect of portals on utilization and efficiency of health systems is still unclear. Patients’ age, gender, education level, computer literacy, and number of comorbid conditions may influence use.

**Conclusions:** Evidence suggests that patient portals improve health outcomes and patient satisfaction. Patient attitudes are generally positive. Portals represent a technology whose benefits for health systems utilization are still unclear.

**Health Policy Implications:** This review informs digital health managers, health policymakers and citizens worldwide about the effects of well-designed patient portals on health and provision of care. Health systems and providers need to put more effort to overcome social, economic and literacy barriers in adoption and use of patient portals.

#### Abstract 30 Is uber’ization of relations possible? Examining the pros and cons of telecare models using poverty-aware theories

##### Shlomit Avni

###### Ministry of Health, IL

**Background:** Digital-care (DC) models have been criticized and simultaneously praised as contributing to the expansion/reduction of health disparities. Poverty is a multidimensional social problem prominent in all developed countries and an important determinant of health. Are DC models a threat or an opportunity to people living in poverty?

**Study Question:** To examine the pros and cons of DC models in mitigating the effects of poverty on health and their contribution to people living in poverty's needs.

**Methods:** A qualitative analysis of 3 approaches focused on the delivery of care to people in poverty (“Pro-poor Health Systems”; “Social Exclusion” and “Poverty-Aware social-work -PPSW) was undertaken. 16 principles were grouped into 3 groups (interpersonal-downstream factors; practical-midstream factors; and social-upstream factors). Both groups and principles underwent examination in order to characterize the challenges and opportunities they insinuate to DC models.

**Results:** DC models can help mitigate numerous practical-midstream barriers including: economic barriers to care as well as hidden expenses such as expenses on transportation; help expand health care organizations’ ability to expand outreach to people in poverty; and provide better accessibility to knowledge and entitlements. Contrariwise, DC models are challenging mainly for interpersonal-downstream factors such as emotional needs, trust and close relations and “symbolic” capital, important factors identified as crucial in PASW approaches. Social-upstream factors such as Social exclusion are also challenging for DC models since they potentially strengthen individualistic patient-oriented approaches rather than socially-oriented ones.

**Conclusions:** Consideration of barriers to care for different social groups, stemming from the use of DC models, should expand to include not only the “usual suspects” such as health literacy and technologic accessibility but also to other interpersonal, ethical, social, cultural and political factors.

**Health Policy Implications:** Systematic examination of DC models for care is needed, especially regarding excluded social groups.

## C: Innovation in health economics, and the economics of healthcare innovation

### Sunday

#### Abstract 31 Mature versus registration studies of immuno-oncology agents: does value improve with time?

##### Omer Ben-Aharon^1^, Racheli Magnezi^1^, Moshe Leshno^2^, Daniel Goldstein^2^

###### ^1^Bar-Ilan University, IL; ^2^Tel Aviv University, IL

####### **Correspondence:** Omer Ben-Aharon

**Background:** A unique feature of immune-oncology agents is the potential for durable survival for a subset of patients, however, this benefit can usually not be seen on the early published data used for regulatory approval. Value Frameworks developed by ASCO and ESMO assess the clinical benefit demonstrated in registration trials. We hypothesize that the proven clinical benefit may change with time, as more mature data is available.

**Study Question:** Objectives: To evaluate the impact of mature data of immuno-oncology agents on ASCO and ESMO scores and to examine the concordance of these frameworks using more mature data.

**Methods:** We reviewed all FDA approvals for immuno-oncology agents between 2011-2017, calculated the ASCO Net Health Benefit (NHB) and ESMO Magnitude of Clinical Benefit Scale (MCBS). We checked which agents fulfill the criteria of being rewarded for durable survival. We assessed the concordance between models using Pearson and Spearman correlation tests. We compared the initial results of studies used for registration to mature follow-up data from the same studies.

**Results:** The FDA approved 27 solid tumor indications for immuno-oncology agents between 2011-2017. The correlation between ASCO NHB and ESMO MCBS was high (0.87-0.93). Mature follow-up data were available for 13 of these indications. In 8 of the mature studies, we found improvement in the grade of ASCO and/or ESMO value frameworks. In 2 cases we found a downgrade in the scale.

**Conclusions:** Despite the different approaches, the high concordance between ASCO-NHB and ESMO-MCBS indicates that both models reward treatments as beneficial for the same immuno-oncology agents. Mature data with longer follow-up reaffirmed most of the findings found in the evaluation at the registration studies stage.

**Health Policy Implications:** ASCO and ESMO value frameworks might be used as supporting tools for reimbursement decisions concerning oncology innovate treatments. Analysis of mature data should be considered for the re-examination of decisions made in previous years.

#### Abstract 262 Quantifying a minimum social value of public health care: a general equilibrium model applied to Israel

##### Erez Yerushalmi^1^, Sani Ziv^2^

###### ^1^Birmingham City Business School, UK; ^2^Academic College of Tel Aviv-Yaffo, IL

####### **Correspondence:** Erez Yerushalmi

**Background:** Countries with universal health care have experienced a rising demand for health care services, without a corresponding rise in public supply. This has led to a debate on whether to increase private health care services - especially in hospitals and second-tier healthcare. Proponents for increasing private health care highlight gains in efficiency and innovation, while opponents emphasize its risk to social welfare. However, the monetary value of these gains and losses is seldom quantified.

**Study Question:** What is the minimum social value of public health care that corresponds to indifference between gains in economic efficiency, with losses to social welfare?

**Methods:** We develop a general equilibrium model that distinguishes between public-private healthcare services and public-private financing. Our approach resembles contingent valuation methods that introduce a hypothetical market. However, it is different because we use numerical simulation techniques to compare a regulated with a simulated deregulated health-labor market. Furthermore, the social value is modeled as a byproduct of health care services. The model is then calibrated to our unique health-focused Social Accounting Matrix of Israel, and simulates the introduction of a hypothetical health-labor market - given that it is heavily regulated in the baseline (i.e., the true situation in Israel today).

**Results:** For mid-point parameters, we estimate the minimum social value at around 18% of public healthcare financing. We furthermore simulate a deregulated health care scenario that internalizes the imputed value of social value and search for the optimal weight of public and private health care provision.

**Conclusions:** When assessing the best type of health care, policymakers should weigh the economic gains of deregulation with the lost social value.

**Health Policy Implications:** We show that well-being may even decrease in cases of over-privatization of health care.

#### Abstract 3 Choosing video instead of in-clinic consultations in primary care: discrete choice experiment among key stakeholders: patients, primary care practitioners and policy makers

##### Irit Chudner^1^, Drach-Zahavi Anat^2^, Karkabi Khaled^3^

###### ^1^Technion, IL; ^2^University of Haifa, IL; ^3^Technion; Clalit Health Services, IL

####### **Correspondence:** Irit Chudner

**Background:** Despite being beneficial for Primary care settings, the adoption of Video-Consultations (VC) instead of traditional in-clinic consultations (I-CCs) is complex and slow.

**Study Question:** Understanding VC vs. ICC choice related preferences of the three key stakeholder groups in primary care: Patients, Primary Care Physicians (PCPs) and Policy Makers (PMs), is crucial for achieving better implementation.

**Methods:** Discrete Choice Experiment (DCE) surveys with 12 choice tasks of two labeled alternatives (VC or I-CC) with four VC vs. ICC attributes most relevant to each stakeholder group was filled by 508 Patients, 264 PCPs and 138 PMs. Choice’ attributes were, for both patients and PMs were: (1) Time to next available appointment; (2) Time in-line before consultation; (3) Relationship to PCP; and (4) Quality of consultation. For PCPs these were: (1) Time in-line before consultation; (2) Patient's self-management ability; (3) Consultation purpose; (4) Quality of consultation. Random effects logit model analysis was used to estimate stakeholders’ preferences for attributes.

**Results:** All experiment attributes were significantly important in choosing VC vs. ICC for patients and PCPs groups. Three out of four attributes were significantly important for PMs. Gaps and similarities were identified between stakeholders in attribute rank orders, trade-offs and probabilities of VC take up. PMs VC uptake was 86%, patients’ preferences suggested that 68% of ICC can be switched to VC and PCPs uptake rates were 30% in cases, where consultation purpose is to diagnose and giving treatment and 48% of consultations, where consultation purpose is a follow-up.

**Conclusions:** Our findings show key stakeholders’ preferences for VC integration. Those preferences should be considered when VC systems are about to be used in primary care to optimize the implementation process.

**Health Policy Implications:** Although there is a stronger preference for I-CC among PCPs and patients, alternative combinations of attribute levels can be used to compensate and reconfigure a more preferred VC service.

#### Abstract 90 The positive influence of AppMedic volunteer’s intervention in the emergency department on patient and family satisfaction

##### Yuval Levy^1^, Moshe Sharist^2^, Doron Garfinkel^1^

###### ^1^Sheba Medical Center, IL; ^2^Yitzhak Shamir Medical Center, IL

####### **Correspondence:** Yuval Levy

**Background:** Overcrowded Emergency Departments (EDs) represents a leading national health problem. The rapidly increasing imbalance between populations seeking help and the available professional teams leads to decreased quality of care (QoC), patient/team's frustration, anxiety, even violence. Facing financial distress, obvious solutions of enlarging the professional teams seem impractical. Therefore, health authorities constantly search for different, cheaper solutions.

**Study Question:** To evaluate whether adding volunteers to ED's professional staff relieves anxiety/frustration and improves patients/family satisfaction.

**Methods:** AppMedic volunteers (practiced medical clowns) were assigned randomly to morning/evening shifts at the Wolfson Medical Center adult ED and accompanied patients/families throughout their stay there (study group). The control group was comparable to patients/families who were treated regularly with no interaction with volunteers. Both groups were interviewed by research assistants (RNs), and filled-in two similar questionnaires, upon admission (expectations) and upon discharge, rating QoC. The questions referred specifically to efficacy, attitudes, and QoC provided by both doctors and nurses and the overall satisfaction of the stay at the ER.

**Results:** Between November 2017-March 2018, 387 patients/families met our inclusion criteria, 112 refused to cooperate. Eventually, 275 participants filled questionnaires: 144 were accompanied by AppMedic volunteers (intervention, study group); the remainder 131 comprised the control group. The groups were comparable in demographic and clinical parameters, and in their responses to the first “expectation” questionnaire. However, for all “discharge” questionnaire parameters evaluated, QoC ratings were significantly higher in the volunteer intervention study group.

**Conclusions:** This study proves that adding trained “non-health professionals” volunteers to ED teams, may decrease patient/family anxiety/frustration and improve overall satisfaction. Interestingly, AppMedic volunteers intervention was associated with significantly increased patients/family satisfaction of doctors/nurses performance, although the latter operated “as usual”.

**Health Policy Implications:** The results may encourage health authorities to further promote this important route of incorporating knowledgeable volunteer into our starving health system.

#### Abstract 190 Minimizing abuse of emergency call center services: is technology the right solution?

##### Maya Siman-Tov^1^, Oren Blushtein^1^, Racheli Magnezi^2^

###### ^1^Magen David Adom, IL; ^2^Bar-Ilan University, IL

####### **Correspondence:** Maya Siman-Tov

**Background:** Unnecessary calls to emergency centers consume valuable time and resources that could be dedicated to patients in genuine need for treatment and transport. Literature regarding rates of malicious or prank calls to the emergency medical system is minimal. Legislation and guidelines regarding harassment calls are lacking or unenforced.

**Study Question:** To estimate and characterize intentional false emergency calls to emergency call centers in Israel and to assess the effectiveness of call-tracking technology implemented at Israel’s national emergency medical service (Magen David Adom - MDA) to deter the problem.

**Methods:** A retrospective interventional study was conducted of all emergency calls received by MDA nationwide, from 2012 through 2016. The daily average number of false and missed calls were calculated. The multivariate analysis compared the average number of these calls based on emergency call center location before and after the technology was implemented in during 2015.

**Results:** In 2016, after implementing specialized call-tracking technology, the number of missed calls decreased to zero and the number of false calls decreased significantly. The average number of false and missed calls was consistently higher in dispatch centers located closer to Israel's borders. Higher numbers of false calls were correlated to missed calls (r=.425, p<.001).

**Conclusions:** In Israel, most false emergency calls originate from areas outside the country and thus, require specialized reactive and proactive technological solutions rather than laws, enforcement or education. Reducing and even eliminating phone harassment, along with fewer missed calls improved the effectiveness of emergency services for the general population.

**Health Policy Implications:** Healthcare professionals and stakeholders are concerned that misuse of emergency call centers stress service provision and may jeopardize patient care. The solution presented here can be implemented by various rescue organizations to reduce inappropriate use. Special technology can reduce and almost eliminate harassment calls without additional staff.

#### Abstract 46 HPV-DNA-Primary screening in Israel is more efficient than cytological screening (PAP TEST)

##### Eduardo Schejter, Einav Shainadman, Talia Feinberg, Judith Sandbank, Noa Triki, Dalia Rabinobich, Jacob Segal

###### Maccabi Healthcare Service, IL

####### **Correspondence:** Eduardo Schejter

**Background:** The primary-cytological-screening for detecting cervical cancer and precancerous lesions has been proven in reducing morbidity and mortality of cervical cancer. In order to improve the sensitivity of the survey, it has been proposed to use HPV-DNA as primary-screening instead. Maccabi Healthcare Services (MHS) (cover 25% of the Israeli-population) started to use HPV-DNA-primary-screening based on HPV-DNA only test (Cobas-test, Roche) at 1/3/18, with triage of cytology and genotyping for the referral to colposcopy.

**Study Question:** To compare the rate of indicated referral to colposcopy and the diagnosis of precancerous lesions based on two primary screening methods in the MHS.

**Methods:** Data was collected for each screening-group based on a centralized computerized system. The screening was done for women aged 25-65. The time periods were 1.1.2017-31.12.2017 for cytology-screening (CS) and 1.3.2018-31.12.2018 for the HPV-DNA-screening (HDS). In the CS group, women with abnormal-cytology (≥LGSIL) or occasionally with ASCUS (atypical squamous cell of undetermined significance) were referred for colposcopy. During the HDS, women that were found HPV 16+/18+ or positive for other High-Risk HPV with abnormal-cytology were referred for colposcopy.

**Results:** 95,137 women with HDS tests during the study period were compared to 112,000 women screened by CS. In the HDS group, 4,091women (4.3%) were referred for colposcopy, of which 2.3% were HPV 16+/18+ and 2.0% positive for other HR-HPV with abnormal-cytology. In the CS era 7,392 women (6.6%) were referred for colposcopy of which 4.1% had ≥LGSIL and the rest (2.5%) had ASCUS. The differences were statistically significant by chi-square analysis (OR=0.64, 95% CI, 0.61-0.66, p-value<0.0001) favoring HDS.In the CS group 1,680 (1.5%) women were diagnosed with CIN II+ (precancerous cervical lesions) vs 1,902(2%) in the HDS (OR=1.34, 95% CI, 1.25-1.43, p-value<0.0001) favoring HDS.

**Conclusion:** HDS decreases significantly the rate of indicated referral for colposcopy, when compared to CS and increase significantly the diagnosis of CIN II+ at the first screening round.

**Health Policy Implications:** HPV DNA screening in Israel if more efficient and preferred than the conventional cytological screening.

#### Abstract 236 Is frontal triage an effective alternative to physician referral-based triage? A prospective cohort study

##### Susanna Mordechay^1^, Chaya Glickman^1^, Hagit Baranes^1^, Leonid Kalickman^2^

###### ^1^Meuhedet Health Services, IL; ^2^Ben-Gurion University of the Negev, IL

####### **Correspondence:** Susanna Mordechay

**Background:** In Israel, physiotherapy-led triage is conducted in patients with musculoskeletal problems (MSKP) referred to physiotherapy clinics (PTC). Patients are classified as “urgent” or “non-urgent” according to the medical information appearing in the doctor's referral.

Currently, “urgent” patients are assessed for initial examination and treatment within 48 hours at Meuhedet PTCs. “Non-urgent” patients are assessed according to availability, at an average of 5 weeks.

Prolonged waiting may cause some patients to use unnecessary medical services and enter the chronic phase of their disease, and therefore impaired functioning.

Research shows that triage methods that enable early physiotherapy intervention, reduce waiting times and usage of medical services and show better clinical results.

**Study Question:** To evaluate whether a Frontal Triage Method (FTM) is an effective alternative to the current physician referral-based triage method.

**Methods:** A trial was conducted in a large PTC. The computer program for triage appointments was adapted and a new triage protocol introduced. All patients were assessed by a physiotherapist within 48 hours and received an initial intervention. Patients were classified as Urgent, Moderately-Urgent, Non-Urgent and continued treatment according to urgency. The following data was collated; waiting time, the number of treatments, clinical results for lower/upper limb MSKP and patient satisfaction. The study period was 11 months and included patients “Before FTM” and “During FTM Intervention”.

**Results:** 2,122 patients were included; 771 before FTM, 1,070 during FTM and 281 were not triaged. Waiting times decreased from 19.98±16.59 to 1.89±1.34 days (p<0.001), the number of treatments decreased from 4.97±3.57 to 4.37±3.49 (p<0.001, (clinical results for lower limb improved (p=0.037), and overall patient satisfaction was maintained.

**Conclusions:** FTM promotes reduced waiting, decreased usage of medical services and is cost effective. FTM effectiveness should be tested in the future in a cluster-wedge trial.

**Health Policy Implications:** Health policy decision-makers should consider implementing a physiotherapy-led triage system and encourage a change in current referral to PT.

### Elevator Pitch

#### Abstract 20 Managing drug shortages - the Israeli experience (2013-2018)

##### Alla Vishkautzan, Eli Marom, Einat Gorelik

###### Ministry of Health, IL

####### **Correspondence:** Alla Vishkautzan

**Background:** The growing number of drug shortages (DS) is of worldwide concern. This problem presents challenges to healthcare professionals and regulatory authorities and may have a direct impact on public health.

Early notification regarding future shortage is essential in order to minimize the potential risks to patients and the health system.

The database of the pharmaceutical division in the Ministry of Health (MoH) was established in 2013, detailing the cause, duration of DS, and the availability of generic or therapeutic alternatives. Since 2017 more detailed causes of the DS are requested.

As from 2016, the MoH instructed the pharmaceutical industry to hold in any time stock of at least 1 month's supply of all registered drugs in Israel.

**Study Question:** Do the mandatory early notification and the obligation of maintaining at least one month's supply of a drug reduce the number of DS in Israel?

**Methods:** Using the database of the pharmaceutical division, between the years 2013-2018.

**Results:** According to the data collected by the MoH, between 2013 and 2018, 1,580 DS notifications were received. In each of these years there was an increase in the number of DS, along with a decline in permanent drug discontinuations, and in immediate notifications. Among the reasons for temporary DS, delay in the delivery of goods was a primary cause (25%).

**Conclusions:** Despite all the steps taken by the MoH and efforts to reduce the scope of DS and their impact on public health, their numbers continue to rise annually albeit with a decline of immediate notifications of permanent market withdrawals.

**Health Policy Implications:** Drug shortages pose a significant hazard to public health in Israel and worldwide. An open dialog between all stakeholders is required in order to minimize the impact of DS. More measures, including the legislation of deterrent measures, should be considered in order to minimize the frequency of DS.

#### Abstract 120 Clinical characteristics and outcome of elderly patients admitted for acute cholecystitis to medical or surgical departments

##### Itamar Feldman, Dvora Shapiro, Lena Feldman, Gabriel Munter, Amos M. Yinnon, Reuven Friedmann

###### Shaare Zedek Medical Center, IL

####### **Correspondence:** Itamar Feldman

**Background:** Acute cholecystitis is a common clinical condition. Even though cholecystectomy is the definitive treatment, in many instances, conservative treatment is both feasible and safe. In our hospital, it is not uncommon to encounter elderly patients with acute cholecystitis in medical and acute geriatric wards.

**Study Question:** Which is the most appropriate admissions’ practice for elderly patients with acute cholecystitis, medical or surgical departments?

**Methods:** A retrospective review of all patients >65 years of age admitted for acute cholecystitis during a 7-year period.

**Results:** A total of 187 patients were detected, 54 (29%) in medical and 133 (71%) in surgical wards. The mean age (±SD) of the patients was 80±7.5 and was higher among those in medical than surgical departments (84±7 versus 79±7) (p<0.05). Patients hospitalized in medical wards had more comorbidity, disability and mental impairment. However, there was no difference in mortality between the two groups, 1 (2%) and 6 (4%) respectively. Independent predictors for hospitalization in medical departments were COPD (OR=9.8, 95% C.I 1.6-59) and the Norton Scale score (NSS; OR=0.7, 95% C.I 0.7-0.8). The impaired mental condition was the only predictor for hospitalization for more than 1 week. The strongest predictor for having cholecystostomy was admission to the surgical department (OR=14.7, 95% C.I 3.9-56.7). Linear regression showed a negative correlation between NSS and length of hospitalization (LOH; Beta=-0.5), for patients with NSS<17 the mean LOH was lower in the medical group.

**Conclusions:** Elderly patients with acute cholecystitis who require conservative management, especially those with severe functional and mental impairment, can be safely hospitalized in medical wards.

**Health Policy Implications:** Our findings suggest a new policy regarding the hospitalization of elderly patients with acute cholecystitis. Medical departments may be an appropriate site for the management of such patients- for their own benefit as well as that of the surgical department.

#### Abstract 194 Nursing assistants - innovative intervention

##### Ahuva Spitz, Gali Weiss, Tzion Tsohar

###### Shaare Zedek Medical Center, IL

####### **Correspondence:** Ahuva Spitz

**Background:** The role of the non-nursing, health care assistant developed primarily to lower health care costs by undertaking perceived non-nursing duties under the supervision of registered nurses. While nursing assistants represent a substantial proportion of the health care workforce and often are the first responders in providing direct patient care, the growth of their role has taken place without proper preparation, or systematic education and training especially in communication skills. This has raised serious concerns, especially with regard to the quality of care.

**Study Question:** Examining an innovative intervention utilizing simulation to train health care assistants, providing them with tools to deal with challenging situations they encounter in delivering direct patient care.

**Methods:** During 2018, data was collected through focused groups with nursing assistants in addition to questionnaires that were filled by nurses and nursing assistants. The results demonstrated a need to address communication between the nurses and the assistants.100 nursing assistants from the medical-surgical words participated in simulations that were specially tailored for them. Prior to attending the simulation center, social workers met with the nursing assistants in small groups allowing them to speak their emotions through cards and guided imagery. The simulations challenged the nursing assistants with difficult patients or family members. At the end of each session, the actor reflected the nursing assistant how he had felt. Each training day concluded with a discussion presenting communication tools for the nursing assistants.

**Results:** The closing questionnaires demonstrate a high level of satisfaction with the intervention.

**Conclusions:** The next planned step is to follow the nursing assistants utilizing qualitative and quantitative analysis focusing on the quality of care through tasks that they perform.

**Health Policy Implications:** The policy will be to provide twice a year a simulation-based training refresher course in order to fully implement a culture of quality and safety amongst nursing assistants applying communication skills.

#### Abstract 212 Budget-impact of drugs for orphan diseases (orphan drugs) in the Israeli health basket: a longitudinal analysis

##### Tal Morginstin^1^, Ariel Hammerman^2^, Noa Triki^3^, Baruch Weinreb^4^

###### ^1^Ministry of Health, IL; ^2^Clalit Health Services, IL; ^3^Maccabi Healthcare Services, IL; ^4^Ben-Gurion University of the Negev, IL

####### **Correspondence:** Tal Morginstin

**Background:** Since the adoption of orphan-drug legislation in the US and Europe, the development of orphan drugs has grown rapidly. The rareness of each disease has led manufacturers to request extremely high prices for such drugs, and therefore their reimbursement has become a significant issue for healthcare decision makers. Although their high cost per patient, the Israeli Health Basket updating committee has tended to recommend accepting most orphan drugs, mainly because of ‘rule of rescue’; saving identifiable patients with life-threatening illnesses, regardless of treatment costs.

**Study Question:** The aim of this study was to evaluate the total budget-impact of reimbursing orphan drugs in Israel in the last two decades.

**Methods:** Although there is no agreed definition of a rare disease, we chose to focus on treatments for diseases with a prevalence of less than 1: 80,000. Budgets were figured in 2019 values. Data was collected from MoH publications regarding the annual Health Basket updates.

**Results:** The first orphan drug added to the Health Basket was Agalsidase alfa for Fabry Disease, in 2002. During 2002-2018, a total of 316 million NIS, 4.4% of the entire budget allocated for all Health Basket updates, was provided for reimbursing 41 novel orphan medicines.

**Conclusions:** We have found that in Israel, although a permissible attitude, the proportion of the total budget allocated for orphan drugs, seems to be tolerable, mainly due to small patient numbers.

**Health Policy Implications:** The price of orphan drugs is a substantial challenge, especially since it has very little to do with incremental benefit. Fears that growth in the availability of novel orphan therapies will lead to an unsustainable cost escalation are currently not justified. However, it cannot be expected that the Health Basket will accept at any price all effective orphan drugs, since numerous significant and cost-effective treatments will need to be forgone for many other patients.

#### Abstract 256 Allocation of resources among medical fields in updating the Israeli ‘health basket’, is there a ‘cancer premium’?

##### Ariel Hammerman^1^, Noa Triki^2^, Tal Morginstin^3^, Baruch Weinreb^4^

###### ^1^Clalit Health Services, IL; ^2^Maccabi Healthcare Services, IL; ^3^Ministry of Health, IL; ^4^Ben-Gurion University of the Negev, IL

####### **Correspondence:** Ariel Hammerman

**Background:** The Israeli ‘Health Basket’ is updated annually according to a government allocated budget. The budget allocated is far from being sufficient to keep up with the ever-growing demands of the healthcare arena, which makes priority setting inevitable. Among health interventions, the cost of cancer treatment has been receiving increased public attention, mainly because of the hardship and short life expectancy associated with the disease and the high cost of the newly developed anti-cancer drugs.

**Study Question:** We attempted to assess how the resources allocated for updating the Health Basket were distributed by the ‘Basket Updating Committee’, between the different medical fields in the last two decades, and whether oncology treatment might be receiving a ‘cancer premium’.

**Methods:** Data was collected from MoH publications regarding the annual Health Basket updates. Budgets were figured in 2019 values.

**Results:** During 1998-2019, resources for new health technologies in the ‘Health Basket’ were allocated to 32 different clinical fields. The therapeutic areas that received the highest budget allocation were: Oncology (33.45%) (Solid tumours (22.49%) and Hemato-oncology (10.96%)), Gastroenterology (7.13%), Diabetes (6.65%), Cardiology (6.59%), Neurology (6.09%), Rare diseases (5.76%) and Pulmonology (3.27%).

**Conclusions:** We have found that in Israel, a third of all resources allocated for new health technologies, were dedicated to cancer treatment, mostly solid tumours. Other diseases received significantly smaller amounts.

**Health Policy Implications:** The access to new expensive anticancer drugs is of concern to patients, decision-makers and the general public. Many reimbursement agencies, such as NICE in England, are still debating whether to place a higher value on end-of-life cancer care. It seems that at least de-facto, decision-makers in Israel have already accepted that cancer treatment deserves a special premium and that patients in Israel should have access to the effective cancer treatments, taking into account the necessity of declining funding for other diseases.

### Monday

#### Abstract 42 The need for differential tariffs in Israel in the era of aging population and emerging technology: cardiac surgery as a case study

##### Joseph Mendlovic, Rachel Tauber, Shuli Silberman

###### Shaare Zedek Medical Center, IL

####### **Correspondence:** Joseph Mendlovic

**Background**: Reimbursement for surgical procedures in Israel does not account for diversity in costs of various procedures. With new and more costly technology coupled with higher risk patients needing more complex surgery, these tariffs may not reflect the true financial burden on caregivers.

**Study question**: Does case mix and complexity of procedures significantly affect cost to justify differential tariffs?

**Objective:** To determine the relative cost of heart surgery as function of predicted risk and complexity of surgery.

**Methods**: Cardiac surgery was taken as a case study. All patients (n=4,409) undergoing cardiac surgery at Shaare Zedek Medical Center between 1993-2016 were stratified according to (1) type of surgery and (2) clinical profile (predicted operative risk according to EuroSCORE). Approximate cost of each group was assessed by the average number of days in ICU and ward multiplied by the respective daily cost as determined by the Ministry of Health, plus the cost of fixed components used in the operating room (manpower and disposables). Cost was evaluated according to these variables. Cost variability was determined using ANOVA.

**Results**: Both increased value of EuroSCORE and type of surgery were directly correlated with cost (p<0.0001): up to 180% increase in cost between low and high risk patients in identical surgery and up to 77% increase with respect to type of surgery. There was up to 330% increase in cost between low risk and low fixed price surgery and high risk patients with high fixed price surgery.

**Conclusions:** Cost of heart surgery is directly influenced by patient profile as well as type of surgery. Modern day technology is more costly yet has become mandatory. Thus reimbursement for heart surgery should be based on differential criteria: clinical risk profile as well as type of surgery.

**Health Policy Implications:** An urgent need for design and implementation of a realistic differential tariff model in the Israeli medical reimbursement system.

#### Abstract 175 An innovative non-economic incentive to increase vaccine adherence among HCWS: cost-consequence analysis and evaluation of sustainability

##### Marcello Di Pumpo^1^, Andrea Tamburrano^1^, Andrea Barbara^1^, Francesca Romana Rolli^1^, Americo Cicchetti^1^, Walter Ricciardi^2^, Patrizia Laurenti^2^

###### ^1^Università Cattolica del Sacro Cuore, Roma, Italia; ^2^Università Cattolica del Sacro Cuore; Fondazione Policlinico Universitario A. Gemelli IRCCS, Roma, Italia

####### **Correspondence:** Marcello Di Pumpo

**Background:** Flu vaccination is effective in preventing seasonal influenza, decreases staff absenteeism by reducing costs resulting from loss of productivity and protects fragile patients from experiencing seasonal flu-related complications. The seasonal flu vaccination coverage among healthcare workers (HCWs) in Italy and in other European countries is too low (approximately 10% during the 2017-2018 influenza season).

**Study Question:** The aim of this retrospective observational study was to test the economic sustainability of an innovative incentive measure consisting in granting one paid hour off-work to all HCWs willing to get vaccinated in our hospital.

**Methods:** We performed a cost-consequence analysis (by using the friction cost method) estimating the per-capita HCWs loss of productivity caused by absenteeism. We calculated the overall HCWs salary per hour to identify the cost of our incentive measure. Finally, we estimated the maximum sustainable amount of paid time off-work grantable without causing financial loss for the hospital, also considering the economic advantage for company to provide flu vaccination to hospital workers.

**Results:** We found a loss of productivity due to absenteeism equal to 237.65€ for each vaccinated worker and 413.78€ for each unvaccinated worker. The difference in per-capita loss of productivity was 176.13€. Considering the incentive measure (an average salary is equal to 36.89€/h) the simulation identified the maximum sustainable paid time off-work to be 4.77 hours (286.5 min).

**Conclusions:** We conclude that our innovative non-economic incentive measure to increase the vaccine coverage among HCWs is sustainable and incrementable.

**Health Policy Implications:** Seasonal flu vaccination is effective in preventing seasonal influenza and its related complications so any useful incentive measure to increase vaccine coverage should be used, especially among HCWs.

#### Abstract 60 Physician remuneration in the age of innovation

##### Leah Wapner, Malke Borow, Baruch Levi, Dotan Simchovitz

###### Israeli Medical Association, IL

####### **Correspondence:** Leah Wapner

**Background:** The considerable changes that have taken place recently in the field of medicine, such as the introduction of telemedicine and the development of Big Data, have had a profound effect on the organization of health services. Their impact is increasingly felt in the financial sphere, especially in the need to change traditional earning models of health professionals, chief among them physicians.

**Study Question:** The aim of this study is to explore the changing nature of physician remuneration models globally in light of innovative technological developments, and to examine the need and feasibility of their adoption in Israel.

**Methods:** The authors conducted a literature review of innovative physician remuneration models. Data were collected from a variety of sources including academic works, official government publications, and media reports, mainly discussing the health services of economically and technologically developed countries. Search engines and databases used include PubMed, Google and Google-scholar, ProQuest, Web of Science, and the OECD online library.

**Results:** Two main themes arose frequently in the review: First, the literature considered propositions to reimburse physicians for video or audio check-ins, even if they do not result in an office visit, in order to allow physicians to practice without establishing an in-person relationship with each beneficiary. Second, the development of Information technology accelerates the expansion of Pay-for-Performance (P4P) models to the point of connecting physician earnings to patient satisfaction of care.

**Conclusions:** Considerable changes in physician remuneration models have been taking place globally in light of profound technological developments. These should be taken into account in the Israeli context.

**Health Policy Implications:** Future negotiations between stakeholders need to include examination and discussion of innovative mechanisms of remuneration, in order to facilitate successful adoption and implementation of technological innovations in medicine. The impact of such mechanisms on the relationship between the patient and the care provider should also be considered.

#### Abstract 191 The 2013-14 expansion of activity-based hospital payment in Israel: an evaluation of the effects on inpatient activity of 15 procedures

##### Ruth Waitzberg^1^, Wilm Quentin^2^, Rina Maoz Breuer^3^, Vadim Perman^4^, Reinhard Busse^5^, Dan Greenberg^6^

###### ^1^Myers-JDC-Brookdale Institute, IL; Ben-Gurion University of the Negev, IL; Technical University of Berlin, Germany; ^2^Technical University of Berlin, Germany; ^3^Myers-JDC-Brookdale Institute, IL; ^4^Ministry of Health, IL; ^5^Technical University of Berlin, Germany; ^6^Ben-Gurion University of the Negev, IL

####### **Correspondence:** Ruth Waitzberg

**Background:** In 2013-14, Israel stepped up the replacement of traditional per-diem payments by Procedure-Related Group (PRG) based hospital payments, a local version of Diagnosis-Related Groups (DRGs). PRGs were created for selected procedures in urology, general surgery, gynecology and ophthalmology.

**Study Question:** How did this change affect inpatient activities, measured by the number of discharges, average length of stay (ALoS), and the case-severity Charlson Comorbidity Index (CCI)?

**Methods:** We investigated the impacts of the PRG-payment reform on 15 procedures. Observations covered groups of inpatients, by age and gender, who underwent these procedures in 2005-2016 at all non-profit hospitals. We examined the effect of the payment change on the number of discharges, ALoS and CCI using a multivariable analysis of Ordinary Least Squares controlling for patients, hospital characteristics, and year fixed-effects.

**Results:** Data on 89,533 patients were examined. During the study period, the ALoS decreased except for one procedure, the number of inpatients increased for most procedures, and case severity remained stable. The multivariable analysis suggests that the transition to PRG-payments contributed to changes in ALoS or case severity for only 3 out of 15 procedures examined. The PRG-reform contributed to changes of 10%-45% in the number of patients, but there was no clear trend: it increased in 9, and decreased in 5. The changes did not follow a clear pattern according to procedures’ price changes after the reform.

**Conclusions:** Factors that may have hampered the effects of the PRG-reform are conflicting incentives created by other co-existing hospital-payment components, such as revenue caps and retrospective subsidies, and the lack of resources to increase productivity.

**Health Policy Implications:** Provider payment reforms should carefully coordinate the entire payment system, otherwise the incentives may be blurred and the reforms may miss their goals.

#### Abstract 267 Markets, medicines and miles: a novel method of measuring access and analysing demand for primary care in complex healthcare markets

##### Benjamin Palafox, Kara Hanson, Catherine Goodman, Dina Balabanova, Martin McKee

###### London School of Hygiene & Tropical Medicine, UK

####### **Correspondence:** Benjamin Palafox

**Background:** In many low- and middle-income countries (LMICs) many common conditions, such as malaria and hypertension, are treated sub-optimally. Addressing such treatment gaps requires a better understanding of the alignment between healthcare systems and patient needs. This is particularly relevant for conditions typically treated by a diverse array of public, private and even informal providers, many of which are often overlooked by common access measures.

**Study Question:** What is the utility of a novel approach of linking comprehensive supply- and demand-side data in order to measure access to healthcare from the household perspective in LMICs?

**Methods:** Survey data from households and the complete range of treatment sources in their vicinities were combined to produce indicators of access for malaria and hypertension from the household perspective in several LMICs. We compare differences in access across urban and rural areas and countries and use these indicators to model various determinants of treatment-seeking behaviour.

**Results:** While free or subsidised treatment for malaria and hypertension is available to many households from public facilities, private providers – including unregulated general retailers – predominate the range of accessible options, particularly for rural households. Private treatment options were on average more expensive. For malaria treatment, provider distance, rather than treatment price was the main provider characteristic that drove treatment-seeking behaviour, while the opposite was observed for hypertension.

**Conclusions:** Examining healthcare access from the perspective of households provides more intuitive and meaningful measures of access in pluralist and varied healthcare markets. This approach also facilitates an understanding of the drivers of changes in access and treatment seeking behaviour, which could help to better explain the performance of complex interventions that to improve healthcare access.

**Health Policy Implications:** Applying the developed methods to measure and analyse access to healthcare provide the improved evidence on the ‘last mile’ coverage of primary care interventions that is needed to reduce important treatment gaps and health inequalities.

#### Abstract 105 Home hospital vs. institutional treatment for sub-acute care patients – an innovative setting impacting health economics

##### Angela Irony, Haled Abu-Hussain, Natali Stein, Nachman Ash

###### Maccabi Healthcare Services, IL

####### **Correspondence:** Angela Irony

**Background:** The increase in life expectancy is accompanied by higher financial and operational loads throughout the health system. One of the possible solutions for easing up these loads is home hospitalization. Maccabi Healthcare Services initiated home hospital setting/alternative for subacute patients.

This research focuses on comparing home hospital and “conventional” hospital care parameters for the sub-acute patient.

**Study Question:** Does home hospital have an economic impact on the health practice compared to institutional treatment?

**Methods:** A prospective cohort study comparing parameters in home hospitalization versus institutional hospitalization. Data was extracted from HMO information systems.

The population includes patients characterized as sub-acute with the following diseases/treatments: Infectious diseases (e.g. p, UTI, skin infection), chronic diseases (e.g. CHF, COPD) and Metabolism Disorders.

**Results:** Since December 2017, 275 patients were recruited to the home hospital setting involving the cooperation of 3 hospitals.

Home hospital patient's data were compared to cumulative total HMO data of 27,215 internal ward hospitalizations (over 3 years).

Total home hospitalization days were 1,089. The average length of treatment is 4.0 (1.9) days in home hospitalizations versus 4.3 (5.4) in institutional hospitalizations. Rehospitalizations within 7 days is 5.9% days in both settings and 30 days rehospitalization is 13.8% in home hospitalization vs. 16.3% in institutional hospitalizations.

Total cost of home hospitalization setting is less than 50% of the comparable alternative of institutional cost. Economic saving is therefore highly significant.

**Conclusions:** Home hospital seem like a comparable alternative to institutional hospitalization with a clear economic benefit.

**Health Policy Implications:** Home hospital care seems a substitute alternative for subacute inpatient care which is conceptually innovative and cost saving. This alternative may be an important option for handling aging population side effects in Israel.

#### Abstract 152 The ministry of health financial incentive program to improve diagnosis and treatment of infectious diseases in nursing homes

##### Ayelet Berg-Warman^1^, Shmuel Be'er^1^, Aharon Cohen^2^, Pinhas Berkman^2^, Yohanan Samuel^2^, Ira Glozman^2^

###### ^1^Myers-JDC Brookdale Institute, IL; ^2^Ministry of Health, IL

####### **Correspondence:** Ayelet Berg-Warman

**Background:** Approximately 2% of Israeli elderly live in Long-Term-Care institutions. When there is an acute worsening of the medical condition of residents of nursing homes (NH), they need medical services that are not usually available in the institution and are referred to a general hospital (GH). Improving the diagnosis of infectious diseases and treating them with antibiotics can reduce the scale of referrals to GHs. The Ministry of Health has therefore begun to authorize NHS to administer IV antibiotics. In 2017, it implemented a pilot to provide financial incentives to the NHS, aimed at reducing the number of residents being referred to GHs.

**Study Question:** To evaluate whether the pilot achieved its goals.

**Methods:** Comparison over time of parameters associated with quality-of-care in NHS participating in the pilot, and comparison between NHS participating and not participating in the program.

The study analyzed administrative data on admissions to GHs and patient deaths, as well as information from interviews conducted with staff.

**Results:**

- Between 2016 and 2017, the scale of referrals to GHs declined (approximately 2,700 hospital days per year). The relatively largest decline was in NHS that participated in the pilot.
- There was a reduction of approximately 4,800 hospital days in the nursing wards and the average hospital stays declined. In contrast, there was an increase of approximately 2,000 hospital days in the mentally frail wards.
- In the institutions participating in the pilot, there was a considerable decline in in-house mortality rate.

**Conclusions:** The pilot contributed to structuring processes of diagnosis and treatment of patients with infectious diseases, thereby reducing the number of referrals to GHs and in-house mortality. This means financial saving while improving several aspects of diagnosis and treatment.

**Health Policy Implications:** It is important to understand the reasons for the increase in the hospitalization rate of mentally frail patients.

#### Abstract 16 An optimal charging scheme for human embryos’ storage service: a source for a secondary market

##### Avi Herbon, Yael Lahav, Uriel Spiegel

###### Bar-Ilan University, Zefat College, IL

####### **Correspondence:** Avi Herbon

**Background:** A growing bank of frozen human embryos is a negative outcome of cryostorage of human embryos as a part of in vitro fertilization treatments. The existence of remaining unused embryos puts the service provider (the hospital) in a fragile situation, especially when the issue of discarding is raised or when unexpected failures associated with preserving the inventory occurs. Moreover, absence of intrinsic incentives to donate embryos to the secondary market, as well as the increased operational costs with time, for keeping the storage service viable, add these unique service additional barriers.

**Study Question:** We develop an optimization model which determines a charging scheme for the couples using the storage service and a payment scheme to couples who agree to give their remaining unused embryos to be a source for the secondary market. Couples who do not agree are charged with an additional discarding payment.

**Methods:** This model is analytically developed and the optimal solution is obtained through optimal solution algorithms. A numerical example and sensitivity analysis of the key parameters is conducted.

**Results:** A comparison of the suggested model with other pricing strategies, including the one which offers the service for free, is presented, while the superiority of the suggested model in increasing service provider's profit and in decreasing unused remaining inventory is demonstrated.

**Conclusions:** This mechanism motivates donations to secondary market and reduces the remaining unused inventory.

**Health Policy Implications:** Optimal pricing scheme in all of our experiments included no more than two-part tariff (associated with the primary market). This, of course, eases the pricing scheme, as well as makes it more convincing in being accepted by the market.

### Elevator Pitch

#### Abstract 101 The impact of child injury on the Israeli economy

##### Orly Silbinger, Naor Porat, Sharon Levi, Esti Golan, Natalie Nir

###### Beterem - Safe Kids Israel, IL

####### **Correspondence:** Orly Silbinger

**Background:** Unintentional injuries of children and youth, from birth to age 17, constitute a heavy economic and health burden on Israeli society. Each year on average 116 children die, over 20,000 are hospitalized and over 200,000 visit emergency rooms due to unintentional injury. There are also long-term economic effects of injury and disability on the child, caregivers and community.

**Study Question:** Evaluate the impact of unintentional child injury on the Israeli economy and the distribution of the burden across different injury types and population groups.

**Methods:** Data on child fatalities, hospitalizations and emergency room visits were collected from national databases for 2008-2016. Data on disabilities was extracted from the Global Burden of Disease database.

Calculations include:

- Direct costs and indirect costs of injury, based on the Human Cost approach.
- Loss of productivity in economic terms related to mortalities and disabilities.
- Analysis by age group, gender, and injury mechanism.

**Results:** The total cost of injury was at least NIS 5.74 billion and NIS 7.46 billion according to the “more stringent” Human Cost approach. Thus, the burden of injury to the GNP for 2016 is 0.5% -0.6%, which includes loss of productivity due to deaths, costs of emergency visits and hospitalization, and loss of quality of life and productivity due to disabilities. Further detailed findings will be presented.

**Conclusions:** Child injury is a serious burden on the Israeli economy. Limitations in this study related to lack of or access to data, such as emergency care visits.

**Health Policy Implications:** This initial analysis is the first and necessary stage of cost-benefit analysis by which effective and focused intervention programs can be selected, priorities and resources allocated effectively, thereby reducing the burden of unintentional injury to children in Israel.

#### Abstract 27 Long term impact from innovations in national health systems: the cases of Brazil and Israel

##### Fabio Gomes

###### Brazilian Chamber of Deputies, Brazil

**Background:** Brazil and Israel are very distinct countries, but both are young multiparty democracies, which adopted universal health systems (UHS) in the late twentieth century.

**Study Question:** The study presents a comparison between the institutions that constitute the UHS of Israel and Brazil, considering the socio-economic context and the health results obtained by the two countries.

**Methods:** The study discusses the characteristics of each health system and compares the evolution of health indicators (on mortality, morbidity, risk factors, health actions, health resources, and health expenditures), from the late 1990’s up to 2018. The data were obtained from the World Bank and the Organization for Economic Co-operation and Development.

**Results:** The tables with health indicators of both countries are available on: https://github.com/DataBrazil/Health_Brazil_Israel/blob/master/Health%20Indicators%20Br_Is_2018.pdf. Generally, the context by the time the health reforms began was already much more favorable for Israel and, currently, the level of advances are still superior to the one of Brazil. However, Brazil has achieved significant results (such as controlling malnutrition and reducing infant mortality) and, in some cases, more favorable data than Israel, such as smoking control and care for people with HIV/AIDS.

**Conclusions:** It is perceived that the Israeli system, which is concerned about achieving greater equity, could benefit from the observation of the successful experiences of the Brazilian system, to avoid the erosion of universality. The Brazilian system, in turn, could benefit from the reflection on strengths observed in the Israeli system, mainly the efficient planning and management of health services.

**Health Policy Implications:** The results of such studies can be used to support proposals for improvements of UHS, even in countries of varying degrees of economic development.

#### Abstract 203 A nationwide policy project to promote consumption of healthy nutrition in Israel

##### Ronit Endevelt, Siegal Sadetzki, Itamar Grotto, Udi Kaliner

###### Ministry of Health, IL

####### **Correspondence:** Ronit Endevelt

**Background:** The increase in the world's population is expected to lead to a radical increase in food production. Extensive production of food raises a challenge of huge innovation processes. The downside of this production is the ultra-processed food that replaces healthy natural products. This may be associated with microbiota changes, may cause obesity and chronic diseases such as Cancer and Diabetes.

To ensure the supply of healthy food, there is a need for a nationwide guidelines regulation and means of enforcement on a national level. Nutritionists are the leading professionals to promote this process, with the collaboration of other sectors such as physicians and food technologists, mediate the regulations to the public.

**Study Question:**

1. How to create national nutrition guidelines that contribute to health, while maintaining sustainability, and equality.
2. How to design methods for implementation and dissemination of the new guidelines.

**Methods:**

1. Establishment of evidence-based nutritional guidelines of a scientific committee.
2. Agreement on regulatory steps to improve the food environment through a committee lead by the Ministry of Health with the participation of stakeholders from many Ministries, the industry, scientists, physicians and nutritionists.
3. Creation of regulations covering various topics that assist to implement the nutritional guidelines in all areas.

**Results:** The Ministry of Health set a few agreements and regulations with the stakeholders:

1. The industry agreed on front of pack labeling and reformulation of packaged foods.
2. The Ministry of agriculture with the ministry of health is formulating the strategic plan of food in Israel.
3. An equality healthy food basket is built with the collaboration of the Ministry of Economics.
4. The Ministry of Education set regulations over healthy food at the lunch program and Kiosks and nutrition education following the Ministry of Health guidelines.

**Conclusions:** The Israeli nutritional guidelines are being published and implemented in all many areas to improve the food environment and eating behavior for better health.

**Health Policy Implications:** The study aims are in health policy and show the way to create national nutrition guidelines that contribute to health while maintaining sustainability, equality, and how to design methods for implementation and dissemination of the new guidelines.

#### Abstract 285 Where to go in an emergency? Determinants of choice of out of hours services in Meuhedet health services

##### Eyal Ariely, Liora Valinsky, Itai Kadim, David Dvir, Roei Bitcher, Anat Toledano, Nuriel Burak

###### Meuhedet Health Services, IL

####### **Correspondence:** Eyal Ariely

**Background:** Out of hours services for medical emergencies are based on three options: emergency departments (ER) in hospitals, in-house emergency clinics (IHC), and emergency clinics run by external providers (EPC). The distribution of the clinics varies across the country. For both clinical and financial reasons it is preferable that members requiring out of hours care attend clinics rather than ER's unless it is strictly necessary.

**Study Question:** What are the demographic, geographic and service-related predictors of the out of hours services used?

**Methods:** We examined all visits to emergency departments (without hospitalization) and clinics during 2018 from Meuhedet data. We analyzed which patient and system related variables, such as age, gender, cultural group, distance from home, district, physician specialization and season were associated with visits to the ER, in-house and external provider emergency clinics.

**Results:** There was no difference in choice of the facility between summer and winter. Ultra-Orthodox members were more likely to attend IHC's than Secular Jews and Arabs (2.2, 1.1 and 0.65) relative to their population proportion. Secular Jews and Arabs were more likely to attend ER's (1.53, 1.73 and 0.93 respectively). Using Multivariate logistic regression, we found that after adjusting for all variables, older age was associated with more ER attendance (OR 1.012 per year, p<0.000), as well as being a member of the Arab population (OR 1.89, 95% CI 1.85-1.93), and having a primary physician who is not a family or internal medicine specialist. In this model, distance from an IHC or EPC was inversely related with attending an ER. Males were slightly more likely to attend ER and women were more likely to attend IHC’ and EPC’s.

**Conclusions:** Although we did not take into account reasons for out of hours visits, it appears that choice of a facility is associated with distance, the expertise of primary physician and culture.

**Health Policy Implications:** In order to reduce unnecessary ER visits, it is essential to understand and address the determinants of customer use and preference.

## Track D: New challenges and threats in the age of innovation –

### Parallel 1

#### Abstract 89 Use of machine learning models for “supervised classification” of legislative proposals related to health in the Brazilian national congress

##### Fabio Gomes

###### Brazilian Chamber of Deputies, Brazil

**Background:** The Brazilian National Congress (BNC) has been outstanding in approving relevant laws related to health policies. However, the monitoring of legislative proposals is hampered by their large numbers. Previous research has identified more than 20,000 proposals submitted between 1999 and 2006 (about 5,000 related to health). Machine Learning can facilitate the task of identifying and classifying the thousands of health-related legislative proposals.

**Study Question:** What is the sensitivity and specificity of machine learning models to predict the classification of health-related legislative proposals in the BNC?

**Methods:** The methodology consists of classifying the health-related legislative proposals that are active in one of the Legislative Houses of the BNC, the Chamber of Deputies (CD); using a typology which has five thematic groups that covers about 60 categories. Between 70 and 80% of those propositions will be used for machine learning, while the rest will be used for the sensitivity and specificity tests of the supervised models, like K-Nearest Neighbors (K-NN), Support Vector Machine (SVM), SVM Kernel, Naive Bayes, Decision Tree and Random Forest.

**Results:** The project has completed its most time-consuming phase: the classification of health related propositions by human classifiers. The data set has 3,592 health related ordinary bills. The most frequent themes were: prevention (35.2%), rights and responsibilities (28.5%), health care (18%) and management and resources (18%). The tests results will be available for presentation in September, 2019.

**Health Policy Implications:** The use of machine learning models has the potential to speed the classification of health-related bills, which can be useful in organizing and monitoring the health agenda in Congress, favoring transparency in the public debate and deliberation on health policies.

#### Abstract 28 Forgotten on the side of the information highway: cognitive barriers to elders engagement with digital technology

##### Shirly Bar-Lev, Daniela Aisenberg, Adi Luria

###### Ruppin Academic Center, IL

####### **Correspondence:** Shirly Bar-Lev

**Background:** E-governance refers to the intensive use of electronic tools and applications for the provision of governmental services. As the use of information and communication technologies intensifies, face-to-face encounters with public service workers (also known as street-level bureaucracy) are replaced by websites and applications. As the relationship between government agencies and the citizen's changes, older adults often find themselves without the skills to benefit from the many advantages of digitation. While much attention has been given to training as a way of improving elders’ digital proficiency, the issue of design has been sidelined within public management research.

**Study Question:** What are the cognitive hurdles that obstruct elders from successfully navigating governmental websites such as the social security website?

**Methods:** We adopted the principles of a ‘think aloud’ methodology to provide detailed participant observations of men and women, ages 71 to 90 years old, as they performed two online tasks concerning entitlements to various health and social benefits. As the participants navigated the online site, we tracked their moves, timed their responses, and asked them to vocalize their thoughts. We were thus able to test their information processing, as well as attention, and memory functions.

**Results:** Despite the participants varied levels of digital proficiency and cognitive abilities (tested using MMSE and digit span tests) they all reported that the completion of the tasks required considerable efforts. The performance was slightly (yet significantly) improved between task 1 and task 2, but their assessments of self-efficacy significantly dropped from task 1 to 2**.**

**Conclusions:** Carefully designed websites are warranted to help elders overcome age-related changes in cognitive functioning.

**Health Policy Implications:** Practical recommendations as to how information should be communicated, to improve senior citizens’ navigation and performance in governmental websites are discussed.

#### Abstract 266 Using digital diaries to research pathways of hypertension care: experiences from engaging with people with hypertension in the Philippines

##### Jhaki Mendoza^1^, Maureen Seguin^2^, Gideon Lasco^3^, Alicia Renedo^2^, Benjamin Palafox^2^, Martin McKee^2^, Dina Balabanova^2^

###### ^1^University of the Philippines, Manila, Philippines; ^2^London School of Hygiene & Tropical Medicine, UK; ^3^University of the Philippines, Diliman, Philippines

####### **Correspondence:** Jhaki Mendoza

**Background:** The rise of digital mobile communication has enabled novel research methods that aim to better understand patients’ experience of treating non-communicable diseases (NCD). Despite this promise, there are important challenges that impede the wider application of such methods. The Responsive and Equitable Health Systems Partnership on Non-Communicable Diseases (RESPOND) study explores these possibilities through the use of ‘digital diaries’ via mobile phone text messaging to track the lived experiences of people with hypertension in the Philippines.

**Study Question:** How effective and acceptable is a digital diary approach to collect prospective longitudinal qualitative data on the lived experiences of seeking care for a chronic illness?

**Methods:** Following in-depth interviews, 40 hypertensive adults were enrolled to submit digital diaries via text message over 12 months. The participants were given weekly mobile phone credit and prompted to share experiences, with entries collated via a dedicated web platform that also permits real-time interaction with researchers.

**Results:** Text messaging was an efficient mode of reaching study participants; however, eliciting relevant information over time required more engagement with researchers. Among the possible explanations for this, one is the participants’ own lack of interest in hypertension as many did not consider it a priority given the absence of symptoms, and view it rather as an intermittent disease. Others include unfamiliarity with the concept of maintaining a diary or reflecting and recording one’s own experiences without the prompting of researchers.

**Conclusions:** Motivating participants to engage with technology-based research methods is a key challenge, and preliminary work to understand participants’ preferences is critical. The ability to maintain two-way interaction between participants and researchers is a feature that holds great potential.

**Health Policy Implications:** Given the ubiquity of mobile phones, the study supports the viability of using text messaging not only for research but also for long-term clinical follow-up and improving adherence.

#### Abstract 12 Physicians’ experiences, attitudes and challenges in a pediatric telemedicine service

##### Motti Haimi^1^, Shuli Brammli-Greenberg^2^, Yehezkel Waisman^3^, Orna Baron-Epel^2^

###### ^1^Clalit Health services; University of Haifa; Technion, IL; ^2^University of Haifa, IL; ^3^Schneider Children's Hospital; Tel Aviv University, IL

####### **Correspondence:** Motti Haimi

**Background:** Telemedicine in general, and telephone triage, in particular, is considered a high-stress clinical activity and may involve stressful situations, and decision making under conditions of uncertainty and urgency.

**Study Question:** We wanted to explore the experiences, attitudes and challenges of the physicians in a Pediatric Telemedicine Service (‘Pediatrician Online service of Clalit’), operating in Israel. We also wanted to explore whether the doctors are using non-medical factors when making clinical decisions in this setting.

**Methods:** We used a qualitative methodology in order to obtain rich data that would reflect the participants’ subjective experiences. Fifteen physicians who are working now or worked during the last 5 years in the ‘Pediatrician Online of Clalit’ service were interviewed individually. Data were analyzed thematically.

**Results:** Seven main themes concerning the difficulties and challenges of the physicians during their work at a pediatric telemedicine service were revealed. These include difficulties diagnosing from a distance, treating unfamiliar patients, working alone, urgency and load of calls, difficulties arising from the technological aspects, and moral conflict between the will to give the parents a good service and the desire to make a proper medicine.

Finally, the physicians described measures they use to overcome the difficulties in the telemedicine setting, enabling them to achieve appropriate decisions. Non-medical /contextual factors were also described to have an effect on their decision-making.

**Conclusions:** The physicians working in the telemedicine setting face various difficulties and challenges. Nevertheless, most of them still reported generally positive experiences, and felt they can give appropriate advises concerning the children's condition.

**Health Policy Implications:** Decision makers should be aware of the challenges the clinicians face in this setting, and the special expertise and training required. Ways must be found to achieve solutions that enable the doctors reaching the correct diagnosis and best possible treatment decisions in this setting.

#### Abstract 171 Socioeconomic position and cancer screening among women in Israel

##### Luz Angela Torres López^1^, Yiska Loewenberg Weisband^2^, Orly Manor^3^, Ora Paltiel^4^, Yael Wolff Sagy^5^

###### ^1^Cardioinfatil Foundation- Institute of Cardiology, Colombia; ^2^The Hebrew University of Jerusalem, IL

####### **Correspondence:** Luz Angela Torres López

**Background:** Screening can improve cancer survival rates, however, population participation in cancer screening is not uniform, with reported inequalities by socioeconomic position (SEP).

**Study Question:** We aimed to study the association between socioeconomic position and cancer screening among women in Israel, and to evaluate whether these associations differed by age and over time.

**Methods:** We used data from the National Program for Quality Indicators in Community Healthcare (QICH), based on electronic medical records from Israel’s four health maintenance organizations (HMO), aggregated by SEP and year. The study population included all adult female Israeli residents whose ages corresponded to screening guidelines during 2002-2017 (N=1,529,233). A four-category area-based measure of SEP was used (ranging from 1 (lowest) to 4 (highest)), obtained from the Central Bureau of Statistics census data and further updated by Points Business Mapping Ltd. Data included screening for colorectal, cervical and breast cancer.

**Results:** Women in lower SEP were less likely to uptake any screening behavior. Greater inequalities were found for cervical cancer screening (OR (SEP 4 vs 1) 3.47, 99.9% CI 3.40 – 3.51) compared to colorectal cancer screening (OR (SEP 4 vs 1) 1.34, 99.9% CI 1.32 – 1.36) and breast cancer screening (OR (SEP 4 vs 1) 1.30, 99.9% CI 1.28 – 1.32). While overall inequalities for breast and colorectal cancer were modest, inequalities were more apparent among older women. Prevalence of breast cancer screening increased over time with a marked reduction in inequalities.

**Conclusions:** Substantial socioeconomic inequalities remain in screening behaviors among Israeli women for all three cancers we assessed but were most pronounced in cervical cancer screening, for which no program exists.

**Health Policy Implications:** While national screening programs exist for breast and colorectal cancer, including reminders and invitations, no such programs exist for cervical cancer. Initiating such programs may help reduce SEP inequalities. The use of large data sets such as the QICH program allows us to identify the need for policies that target improved access to screening for women in lower SEPs.

#### Abstract 148 Using individual-level geographic data to uncover socioeconomic status-independent spatial clusters of mammography adherence in Geneva (Switzerland)

##### José Luis Sandoval, Rebecca Himsl, Jean Michel Gaspoz, Stéphane Joost, Idris Guessous

###### Geneva University Hospitals, Switzerland

####### **Correspondence:** José Luis Sandoval

**Background:** Individual behaviour and health-related outcomes are influenced by the local physical and social environment. It remains undetermined if the environmental influence is independent of individual socioeconomic status.

**Study Question:** We aimed to determine the spatial distribution of mammography adherence in a Swiss urban population (Geneva, Switzerland) and evaluate how independent it was from socioeconomic status (SES).

**Methods:** We used geo-referenced individual-level data (n = 5,002) from participants in the population-based cross-sectional Bus Santé study. We calculated local indicators of spatial association (LISA) and assessed the spatial dependence of mammography adherence. Reported spatial clusters are unadjusted; adjusted for individual educational attainment and neighborhood income; and demographic variables (Swiss nationality and age). We also evaluated the association between adjusted clusters and the proximity to the nearest screening center.

**Results:** Mammography adherence was not randomly distributed in Geneva. Spatial clusters coincided with known SES distribution. Adjustment for SES indicators reduced spatial clusters to 56.2% of their initial size (n = 1,033). Further reduction in individuals exhibiting spatially-dependent behavior (36.5% of the initial size) was observed after adjustment for age and nationality. Proximity to the nearest screening center was not related to the identified SES-independent spatial hot-spots and cold-spots of mammography adherence.

**Conclusions:** Demographic and SES factors shape the spatial distribution of mammography adherence. However, the persistence of spatial clusters after adjustment for these confounders indicates that additional neighborhood-level determinants are influencing the spatial variation of mammography adherence.

Further studies to identify these additional local determinants could lead to targeted public health interventions to improve population health outcomes.

**Health Policy Implications:** Spatial analysis of individual data is a powerful tool to characterise population behavior and identify spatial clusters that are determined by individual behaviors rather than by pre-determined administrative units (e.g. neighborhood and postal code). Taking into account high definition spatial distribution studies has the potential to improve current data-driven health policymaking significantly.

#### Abstract 137 Exploring socioeconomic disparities in diabetes quality indicators in Israeli adults

##### Yael Wolff Sagy, Michal Krieger, Orly Manor, Ronit Calderon Margalit

###### The Israel National Program for Quality Indicators in Community Healthcare; The Hebrew University in Jerusalem, IL

####### **Correspondence:** Yael Wolff Sagy

**Background:** Diabetes Mellitus (DM) is associated with micro- and macro-vascular complications, resulting in a high burden of morbidity and mortality. Populations of low socioeconomic position (SEP) in various countries were found to have increased prevalence of the disease, worse glycemic control, and increased complications.

**Study Question:** To explore socioeconomic disparities in diabetes quality indicators among Israeli adults.

**Methods:** The Israel National Program for Quality Indicators in Community Healthcare obtains data from electronic medical records from the four health plans, covering the entire civilian population. In 2017, 497,397 individuals aged >18 years were identified with DM. Diabetes prevalence, quality of care indicators, including process and intermediate outcomes were explored. SEP was determined on a scale of 1 (lowest) to 10 (highest) according to residential addresses, grouped and classified by the Israel Central Bureau of Statistics into geographical areas, and further refined by a commercial company (Points Business Mapping Ltd).

**Results:** The prevalence of DM in Israeli adults in 2017 was 9.7%, showing a strong SEP gradient, with higher prevalence in individuals of lower SEP when stratified by age-groups. No SEP disparities were observed in process indicators with overall rates of hemoglobin A1c (HbA1c) documentation of 90.9%, ophthalmologic examinations of 72.5%, and kidney function examinations of 92.5%. However, strong SEP disparities were observed in the prevalence rates of uncontrolled diabetes, where the overall rate of HbA1c≥9% was 10.0%, and a 5.4-times higher rate was seen in diabetics of the lowest SES level (23.5%) compared with the highest SEP level (4.3%). Diabetes control had an overall rate of 69.7% and was 1.7-times higher in diabetics of the highest SEP level compared with the lowest SEP level.

**Conclusions:** These findings suggest that access to care does not explain SEP disparities in diabetes control in Israel.

**Health Policy Implications:** Current social benefits policy, compensating patients with uncontrolled diabetes may contribute to the observed gap.

### Parallel 1

#### Abstract 295 Digitizing United States air force medical standards to create a decision-support tool

##### Colby C. Uptegraft^1,2^, Matthew G. Barnes^2^, Jonathan Bickel^1^, Adam Wright^3^, Jonathan D. Hron^1^

###### ^1^Boston Children’s Hospital, Boston, MA; USA; ^2^United States Air Force, Wright-Patterson AFB, OH, USA; ^3^Brigham and Women’s Hospital, Harvard Medical School, Boston, MA, USA

####### **Correspondence:** Colby C. Uptegraft

**Background:** Medical readiness is the ability of military members to safely execute their occupational requirements. To assess readiness in the United States Air Force (AF), clinicians crosscheck each member’s diagnosis(es) and occupation(s) with eight separate documents, an inefficient and error-prone process. Additionally, these documents are not linked or mapped to any medical ontology. Linking these documents and mapping them to ICD codes within a decision-support tool could reduce the administrative burden on clinicians and improve readiness.

**Study Question:** Can AF medical standards be integrated and mapped to ICD codes to create a highly sensitive decision-support tool?

**Methods:** Each standard within the AF Medical Standards Directory was mapped to zero or more ICD-10-CM codes and one or more AF occupational codes. ‘Waiverable’ diagnoses, according to the AF Waiver Guide, were flagged in the mapping. These mappings were validated by two authors (CU, MB) and converted into a SQL database. An RShiny web application was then built as the front-end interface. To validate the tool, a cross-sectional study will compare the tool against an independent SME review. SMEs will review a random sample of 200 outpatient encounters and determine if the encounters justified duty, deployment, and/or fitness restrictions.

**Results:** Overall, 646 of 704 (91.8%) medical standards were successfully mapped to ICD codes. Remaining standards were too vague to identify any matching codes. Additionally, an overview of the methodology used to create this decision-support tool and its validation results will be presented. Measures will include sensitivity, specificity, positive predictive value, and negative predictive value for each restriction category.

**Conclusions & Health Policy Implications:** Creating a readiness decision-support tool could decrease administrative time for clinicians, improve occupational dispositions, and ultimately increase medical readiness. Longitudinal tracking of medical diagnoses and standards violations across occupations using this tool would allow for data-driven adjustments to accession, retention, and standards policies of the United States Armed Forces

#### Abstract 300 Innovation and the Israel journal of health policy research (IJHPR)

##### Bruce Rosen^1^, Avi Israeli^2^, Stephen Schoenbaum^3^

###### ^1^Myers-JDC-Brookdale Institute, IL; ^2^Hadassah Medical Center-The Hebrew University, IL; ^3^Josiah Macy Jr. Foundation, US

####### **Correspondence:** Bruce Rosen

The Israel Journal of Health Policy Research (IJHPR) is both an innovative platform and a platform for innovation.

The IJHPR is a peer-reviewed, on-line, open access journal that is sponsored by Israel’s National Institute of Health Policy. Within just 2 years of its launch in 2012, the IJHPR was already accepted into the prestigious Web of Science – primarily because of its innovative positioning as a journal which is simultaneously national and international. This innovative positioning has also contributed to annual growth of over 20% in both submissions and publications, and to the IJHPR being ranked among the top half of public health journals just 6 years after its launch date.

The IJHPR has also served as a platform for numerous innovations, including:

- Sharing with the international community information about Israeli innovations in public health, health policy, health care delivery, and more
- Enhancing the impact of empirical studies by Israeli scholars via commentaries by leading scholars from abroad – including 18 commentaries from scholars based at Harvard and one commentary by a Nobel laureate in economics.
- The development of a new genre of articles for Israel, which is less of a natural fit for more general policy journals: broad policy analyses focused on major challenges facing Israeli health care
- The use of dynamic, constantly growing, article collections in such fields as digital health, pharmaceutical policy, and health care equity, to highlight and promote areas of excellence in Israeli health care and Israeli health services research
- The ability to share the essence of major Israeli conferences such as this one with a broader audience

While the IJHPR has already contributed significantly to innovation, we feel that it has significant potential to contribute more, and in new ways, in the years ahead. Conference participants are sincerely encouraged to send to the editors (ijhpr2@gmail.com) innovative ideas for how the IJHPR can contribute even more to health care in Israel and beyond.

#### Abstract 289 Disrupting health services research in the us: need, opportunities, and risks

##### Michael Gluck, Danielle DeCosta, Maria Gonzalez, Rachel Dungan, Thomas Blount, Lisa Simpson

###### AcademyHealth, USA

####### **Correspondence:** Michael Gluck

**Background:** To improve the relevance and timeliness of health services research (HSR) in the U.S., AcademyHealth and the Robert Wood Johnson Foundation in the US launched the Paradigm Project in 2019 to identify and test innovative and potentially disruptive changes to the research ecosystem using the tools of “design thinking”. As a first step, AcademyHealth conducted key informant interviews with the project’s Steering Council of established and emerging leaders in and beyond the field.

**Study Question:** What are the most important challenges and opportunities for HSR and AcademyHealth’s Paradigm Project?

**Methods:** AcademyHealth staff conducted semi-structured 45-minute key informant interviews with 3 Co-Chairs and 25 Members of the Steering Council charged with providing guidance to the Paradigm Project. We used qualitative research software and an inductively-developed codebook to thematically analyze interview transcripts.

**Results:** Informants most commonly pointed to incentives in university culture, promotion/tenure standards, peer-review, and academic publishing as significant barriers to the relevance of HSR results for the lived experiences of patients, families, and communities and for health care policy and practice. They also view broader societal trends including new sources of data, increased computing capabilities, and growing expectations for transparent institutions and greater social returns to social investments as providing both opportunities a and mandate to change the way HSR is conducted, disseminated, and used.

**Conclusions:** There is an emerging awareness among established and emerging HSR leaders of an urgent need for an HSR enterprise that supports research using new data and methods, better connects researchers to the systems they research, more effectively moves findings into policy and practice, and rewards researchers for these efforts.

**Health Policy Implications:** This is an opportune moment for changes to the HSR ecosystem that can improve the relevance and timeliness of evidence health policy and practice.

#### Abstract 258 Implementation of an integrated nurse-led care program for community-dwelling older adults in Canton Baselland: the inspire project

##### Mieke Deschodt^1^, Eva Blozik^2^, Matthias Briel^1^, Nicole Probst-Hensch^3^, Matthias Schwenkglenks^1^, Andreas Zeller^1^, Sabina De Geest^1^

###### ^1^University of Basel, Switzerland; ^2^Helsana, Zürich, Switzerland; ^3^Swiss Tropical and Public Health, Switzerland

####### **Correspondence:** Mieke Deschodt

**Background:** In 2018 a new legal framework to redesign community care for older people was approved in Canton Baselland, Switzerland: 86 municipalities will merge into 7 care regions. Each region needs to implement an integrated care concept for older people including a nurse-led information and assessment center.

**Study Question:** INSPIRE aims to implement an integrated community care program for senior citizens in Canton Baselland, and evaluate the success of the implementation (e.g. reach, fidelity) and the impact at patient-, provider- and health systems level (e.g quality of life, provider satisfaction, hospitalization).

**Methods:** INSPIRE is a hybrid type 1 mixed-methods effectiveness-implementation design combining clinical effectiveness evaluation while collecting information to inform implementation in a large quasi-experimental before-after study. The Baselland Older Population Survey (BOPS) will be sent to all 29,000 people aged 75 years and older to map their needs and preferences and will serve as a cantonal baseline measurement.

**Results:** A contextually-appropriate adapted care program based on the results of the systematic literature review and input of the Baselland stakeholders has been developed. The INSPIRE care program includes risk stratification of the older population, nurse-led comprehensive geriatric assessment, care coordination of health and social services, and tailored follow-up. This care program is will be implemented and processes and outcomes will be evaluated in two care regions.

**Conclusions:** INSPIRE will inform the implementation of a new care model by involving stakeholders in co-creating a care concept that is based on scientific evidence, is feasible and acceptable and fits the local context.

**Health Policy Implications:** The integrated care model has the potential to be scaled up as stakeholder involvement is present throughout the project and in-depth process evaluations will be conducted. The INSPIRE project can become a blueprint for other Swiss or international regions to find innovative contextually adapted solutions for aging.

#### Abstract 26 Variations in infant and childhood vitamin D supplementation programs across Europe and factors influencing adherence

##### Ardita Kongjonaj^1^, Suma Uday^2^, Magda Aguiar ^3^, Ted Tulchinsky ^4^, Wolfgang Högler^3^

###### ^1^Prime Ministers’ Office, Albania; ^2^Birmingham Children’s Hospital, UK; ^3^University of Birmingham, UK; ^4^The Hebrew University of Jerusalem; Ashkelon College, IL

####### **Correspondence:** Ardita Kongjonaj

**Background:** Nutritional rickets is a growing global public health concern despite existing prevention programs and health policies.

**Study Question:** What are the differences between infant and childhood vitamin D supplementation policies, strategies and practices across Europe and explore factors influencing adherence?

**Methods:** Representatives of the European Society for Paediatric Endocrinology and other European specialists completed a questionnaire relating to country-specific vitamin D supplementation policy and child healthcare programs, socioeconomic factors, policy implementation strategies, and adherence. Kendall’s tau-b correlation coefficient was used to assess the effect of individual factors on adherence.

**Results:** Responses were received from 29 of 30 European countries (97%). Ninety-six percent had national policies for infant vitamin D supplementation in place. Supplements are commenced on day 1-5 in 50% (14/28) of countries, on day 7-21 in 46% (13/28); only the UK starts supplements at 6 months. Recommended duration of supplementation varied widely from a minimum of 6 months to lifelong in at-risk populations. Good (≥80% of infants), moderate (50-79%) and low adherence (<50%) was reported by 61% (17/28), 28% (8/28) and 11% (3/28) of countries, respectively, with the UK reporting the lowest adherence to supplements (5-20%). Factors significantly associated with good adherence were universal supplementation independent of the mode of feeding (p=0.02), financial family support (p=0.04); monitoring adherence at recommended child care visits (p= 0.001) and the total number of factors (n=11; p<0.001).

**Conclusions:** Good adherence to infant vitamin D supplementation is associated with relatively simple factors such as offering universal supplementation, financial family support and monitoring adherence at recommended child care visits. Implementation strategies matter for delivering efficient prevention policies.

**Health Policy Implications:** There is a call for a more political effort to invest in the implementation of efficient supplementation and fortification programmes to prevent symptomatic vitamin D deficiency, and thereby protect the most vulnerable members of society and minimize inequalities among socioeconomic groups and ethnic minorities.

#### Abstract 160 Making sense of data – building bridges between biostatisticians and policymakers

##### Richard Dale

###### Gothenburg City, Sweden

**Background:** The wellbeing of the population can be affected if policymakers cannot understand that the health of the population is determined by the physical environment, inhabitants’ living conditions and available services. A lot of data is collected, and a lot of register-based studies are published in scientific papers; however, seldom this data makes sense to policymakers.

**Study Question:** The aim of this project was to develop a social surveillance instrument suitable for policymakers.

**Methods:** Sociodemographic and health-related data were collected at the city-level, district-level and subdistrict-level. Maps showing services, housing, roads and public places were produced at subdistrict-level. A policymaker-friendly layout was created in 2014 for one of the ten districts of the city of Gothenburg. Since then the instrument has been updated every year.

**Results:** Knowledge regarding the geographical differences in social determinants and health in the population has increased among policymakers in the intervention area. Policymakers in the selected district use the created instrument in their official meetings while discussing, planning and following up political decisions. Other organizations also have used the created instrument in discussions regarding social and health-related interventions and regarding the allocation of services, recreational areas and new residential areas.

**Conclusions:** The gap between biostatisticians and policymakers can be reduced using the adequate channels and languages.

**Health Policy Implications:** Policymaker-friendly instruments can improve political decisions and improve the health of the population.

#### Abstract 305 Challenges and threats in an era of innovation in health professions education and collaborative practice

##### Stephen C. Schoenbaum

###### Josiah Macy Jr. Foundation, US

Health care needs of people in high and middle income countries have been evolving. The populations of these countries have increasing numbers of persons with multiple chronic conditions and/or complex care problems. Though in part this reflects growth in the numbers of elderly, it involves persons of all ages.

For a long time, the majority of health care services has been delivered in ambulatory settings, and in recent years more complex services can be delivered in ambulatory settings. Yet, the education of health professionals, particularly physicians and nurses, has remained largely in inpatient settings.

Not surprisingly, the education of all health professionals, including physicians, nurses, pharmacists, and allied health professionals, needs to change to meet current and future population needs. This is occurring in several countries. It has been advanced by a number of innovations, particularly development of more interactive teaching and competence based education; simulation-based education and training; and interprofessional education. Each of these innovations presents opportunities and also encounters barriers.

This presentation will discuss several of the following:

- Current levels of competency of graduating medical students and nursing students
- Methods of assessing competence and stimulating improvement
- Mastery learning of skills
- Development of faculty to address adult learning principles
- Development of faculty for interprofessional education
- The economics of ambulatory teaching

It will also address the need for more physicians and nurses for an expanding and aging population in Israel and how Israel might best cope with a seemingly inadequate supply of hospital-based clinical facilities for training the needed professionals.

#### Abstract 170 Overdose from prescription opioids in adolescence, 1999-2017

##### Oren Miron^1^, Kun-Hsing Yu^1^, Rachel Wilf-Miron^2^, Isaac S. Kohane^1^

###### ^1^Harvard Medical School, US; ^2^The Gertner Institute for Epidemiology and Health Policy Research, IL

####### **Correspondence:** Oren Miron

**Background:** Opioid prescriptions increased in the U.S. from 1999 to around 2009, which lead to an increase in overdose deaths from prescription opioids. Since then, there has been a decrease in opioid prescription, including in adolescence.

**Study Question:** What is the trend in overdoses from prescription opioids in adolescents, and does it differ from young adults.

**Methods:** We searched the CDC mortality database for ages 10-19 years (adolescents) and 20-29 years (young adults). We extracted ICD-10 counts for overdose from prescription opioids (ICD-10 codes: X40-X44, X60-X64, X85, Y10-Y14; multiple-causes T40.2). We analyzed the trends for each age group from 1999-2017 using Join Point-Regression (NIH-Version 4.7).

**Results:** At ages 10-19 years, the overdose rate from prescription opioids increased from 1999-2008 by an annual percent change (APC) of 15.12% (95% confidence interval, 9.6% to 20.9%). The rate decreased from 2008-2017 by APC of -5.8% (95%-CI, -9.5% to -1.9%). In 2017 the overdose rate per 100,000 at ages 10-19 years was 0.34 (95%-CI, 0.28-0.39).

At ages 20-29 years, the overdose rate from prescription opioids increased from 1999-2010 by an annual percent change (APC) of 15.18% (95%-CI, 13.3% to 17.1%). There was an increase from 2014-2017 by APC of 13.8% (95%-CI, 5.2% to 23.0%). In 2017 the overdose rate per 100,000 at ages 20-29 years was 4.62 (95%-CI, 4.43-4.82).

**Conclusions:** Our analysis shows that age 10-19 years had a decreasing trend from 2008-2017, while age 20-29 years had a non-significant decrease from 2010-2014, followed by a significant increase. The large decrease in adolescent overdoses might stem from the reduction in an opioid prescription from around 2009. The lack of a decrease in young adults may stem from them receiving opioids as adolescents before 2009 and becoming addicted. Another explanation might be adolescent-specific restrictions.

**Health Policy Implications:** Future studies should also examine the effect of specific restrictions on opioid prescribing in adolescence and sequelae in adulthood.

#### Abstract 17 The health of “Answer Seekers” – addressing the needs of young Israelis moving from the ultra-orthodox to the secular community

##### Ronit Pinchas-Mizrachi^1^, Baruch Velan^2^

###### ^1^Ramat-Gan Academic College, IL; ^2^The Gertner Institute for Epidemiology and Health Policy Research, IL

####### **Correspondence:** Ronit Pinchas-Mizrachi

**Background:** Many young Israelis leave the Orthodox religious community to join the mainstream non-religious community. These groups often referred to as “Answer-Seekers” are gaining volume in the Israeli society both in number and visibility. The transition process could be very strenuous on the individual and result in undesired effects on wellbeing and health. Moreover, their emerging health needs could be jeopardized by inherent barriers in access to healthcare.

**Study Question:** To examine the health consideration of “Answer-Seekers” in an attempt to define their vulnerabilities and needs, and define measures for improving accessibility**.**

**Methods:** 12 young adults who have recently made the transition were asked to relate to health problems bothering the community and to accessibility barriers. The semi-structured interviews were analyzed qualitatively.

**Results:** Interviewees indicated that the “Answer-Seekers” population could be affected by mental health problems, including stress and depression, by sexual health problems related to unsafe practices, and by risks related to substance abuse and hazardous behavior. Interviewees suggested that these problems are associated with difficulties encountered prior to and during the post-transition process. Quest for help is often hampered by health illiteracy, stigmatization of mental and sexual vulnerabilities and prejudice.

**Conclusions:** The “Answer-Seekers” population is a newly developing community with specific health needs. Comparison to findings regarding the health of immigrants, and LGBT populations suggest that the process of transition per se could trigger health problems.

**Health Policy Implications:** Health authorities are urged to pay attention to the problems of this emerging group and provide appropriate health measures.

